# The Candela and Photometric and Radiometric Measurements

**DOI:** 10.6028/jres.106.007

**Published:** 2001-02-01

**Authors:** Albert C. Parr

**Affiliations:** National Institute of Standards and Technology, Gaithersburg, MD 20899-8440

**Keywords:** cryogenic radiometer, laser power, photodiodes, photometry, radiation temperature, radiometry, standards

## Abstract

The national measurement system for photometric and radiometric quantities is presently based upon techniques that make these quantities traceable to a high-accuracy cryogenic radiometer. The redefinition of the candela in 1979 provided the opportunity for national measurement laboratories to base their photometric measurements on optical detector technology rather than on the emission from high-temperature blackbody optical sources. The ensuing technical developments of the past 20 years, including the significant improvements in cryogenic radiometer performance, have provided the opportunity to place the fundamental maintenance of photometric quantities upon absolute detector based technology as was allowed by the 1979 redefinition. Additionally, the development of improved photodetectors has had a significant impact on the methodology in most of the radiometric measurement areas. This paper will review the status of the NIST implementation of the technical changes mandated by the 1979 redefinition of the candela and its effect upon the maintenance and dissemination of optical radiation measurements.

## 1. Introduction

**Fig. 1:**
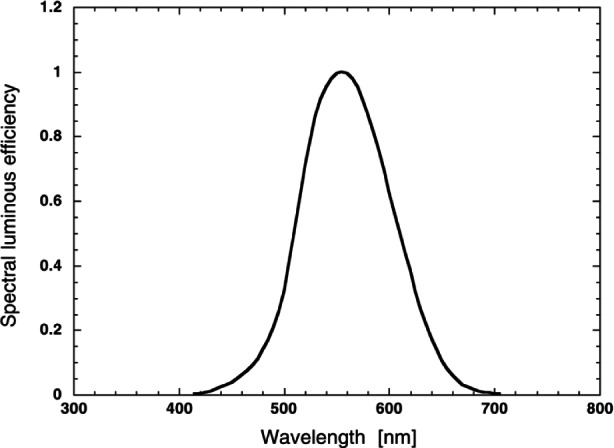
CIE (International Commission on Illumination) visual luminous efficiency distribution for photopic vision. The function is normalized to unity at 555 nm. The horizontal axis is wavelength of light.

The science of performing light measurements accounting for human visual response is referred to as photometry. Photometric quantities, such as luminous flux [International System of Units (SI) unit lumens], are designed as a realistic metric for human perception of light by appropriately accounting for the human eye’s varying response as a function of wavelength. Human visual response decreases its sensitivity at short wavelengths in the blue and at long wavelengths in the red and reaches its greatest sensitivity in the green region of the spectrum. Based in part upon work by Gibson and Tyndall at NBS in 1923, the International Commission on Illumination (CIE) defined an average spectral response for the human eye for well-illuminated conditions, which is termed the spectral luminous efficiency for photopic vision. This spectral response is usually denoted as *V*(λ) in recognition of the fact that this distribution was originally called the “visibility curve” [1, 2]. This international standard for spectral luminous efficiency is shown in Fig. 1. Inspection of this distribution indicates that the human visual response peaks at about 555 nm and decreases to zero in the ultraviolet (UV) and the infrared (IR) wavelength regions. By definition the response has been set to unity at the peak of the response at 555 nm. Different response functions are used as standards for the spectral luminous efficiency for lesser illuminations, which are called mesopic vision for intermediate levels of illumination and scotopic vision for very low levels of illumination. These matters are not central to the discussion here and hence will not be expanded upon. The interested reader is referred to the literature for the details and applications of these definitions [3, 4].

Prior to 1948, there was no widely agreed upon international standard for luminous intensity (roughly, the perceived brightness of a light source). The international units for luminous intensity and other photometric quantities were maintained by comparisons of artifacts, including candles, lamps, and other types of light sources, none of which were based on fundamental physical laws. In 1948, the General Conference on Weights and Measures (GCPM) adopted a definition of the unit of luminous intensity, called the “new candle,” derived from the radiation properties of platinum at the temperature where it froze from a liquid to a solid. This definition, for the first time, based photometry on a repeatable physical process or phenomena that could be independently recreated and observed anywhere in the world. The 1967 CGPM changed the name of the unit of measurement for luminous intensity from “new candle” to “candela” but did not change its physical definition, hence the following definition was used for the candela from 1948 to 1979 [5]:

The candela is the luminous intensity, in the perpendicular direction, of a surface of 1/600000 square meter of a blackbody at the temperature of freezing platinum under a pressure of 101325 newtons per square meter. (13th CGPM, 1967, Resolution 5).

Most national laboratories found it difficult and expensive to maintain a platinum freezing-point blackbody and a consensus developed to change the definition of the candela to one based solely upon the measurement of optical power [6]. In 1979 the CGPM adopted a recommendation from the International Committee on Weights and Measures (CIPM) and redefined the candela in terms of a specific amount of optical power:

The candela is the luminous intensity, in a given direction, of a source that emits monochromatic radiation of frequency 540 × 10^12^ hertz and that has a radiant intensity in that direction of 1/683 watts per steradian. (16th CPGM, 1979, Resolution 3)

The specified frequency was chosen to be at the peak of the *V*(λ) distribution (555 nm) and the factor 1/683 watts per steradian was chosen based upon the best estimate at that time of the freezing point of platinum. These choices allowed continuity between the new definition and the old one. This definition also decoupled the photometric units from the radiation properties of a source and allowed the photometric units to be determined by the measurement of optical power with appropriate photodetectors.

It might appear that the definition of the candela only pertains to light at a wavelength near 555 nm and thus a monochromatic light source must be used to determine the candela. The central point of photometry, however, is to measure all light according to human visual perception. To that end, the CIPM adopted the CIE definition for spectral luminous efficiency *V*(λ) to determine luminous intensity at other wavelengths on a relative basis.

This extension of the definition also provides a practical prescription for measuring photometric units by using optically filtered photodetector systems. These photodetectors are constructed to have a wavelengthdependent response that is closely proportional to the *V*(λ) distribution. In Sec. 2 of this paper we will discuss the foundation of detector-based metrology, and in Sec. 3 we will show how filtered photometers provide methods for determining photometric quantities according to the candela definition within SI.

Table 1 provides a listing of photometric quantities and their radiometric counterparts. For historical reasons the fundamental photometric quantity in SI has been luminous intensity which, although it is a property of a light source, can be related to detector quantities by measuring the luminous flux of a source under conditions of known geometry. Luminous flux, measured in lumens, is a more commonly used quantity in photometry. In illumination applications, where a lamp fixture is present that might include reflectors and diffusers, the luminous flux of all the light emitted by a lamp in all directions is a key measure of the lamp’s performance. The number of lumens produced by a lamp proportioned to its electrical power consumed is a figure of merit for the lamp’s efficiency.

## 2. Absolute Detector Radiometry

The 1979 redefinition of the candela was a major driving force in radiometry and photometry that spurred the need for, and subsequent development of, improved optical radiation detectors in the visible wavelength region. While light sensors such as photomultiplier tubes and cadmium sulfide devices were well known, such technologies were not suitable as fundamental standards. Fortunately, at the time the redefinition was formulated, technology had progressed. Newer types of photodetectors allowed photometry and radiometry to become detector-based, rather than based on traditional thermal source techniques.

By the 1970s, silicon photodiodes had become available with unprecedented stability [7]. Advances in semiconductor technology provided a wealth of new types of solid-state photodetectors, both from silicon and other materials. Solid-state devices are particularly attractive as sensors since they can directly supply an electrical signal, usually current, which can be proportional to the input optical signal over many orders of magnitude. Their internal impedance and other electronic characteristics lend themselves to easy mating with modern solid-state electronics and thereby provide a high-quality and inexpensive sensor system for many applications of optical radiation measurement.

At about the same time silicon devices were being perfected for use in radiometry, better electrical-substitution radiometers were also developed. These included cryogenic devices operating near liquid-helium temperatures with a relative combined standard uncertainty of 0.01 % or better. Electrical-substitution radiometers are constructed by devising an absorbing receiver that collects optical power and as a result undergoes a temperature rise. The optical power is determined by using electrical power to produce the same temperature rise or, in most practical implementations, to maintain a steady-state temperature as the optical power load varies. The equality of the electrical and optical power is implied because of their equivalent thermal effect on the system.

Electrical substitution devices are often referred to as absolute detectors because they determine the radiant flux incident upon them by direct reference to physical laws and do not depend upon another optical power-measuring device for their calibration. The presumption of the equivalence of electrical power and optical power heating of a system is verified by careful characterization of the radiometer. Similarly, in the case of silicon, the knowledge of the semiconductor physics is thought to be adequate for describing the internal quantum efficiency and hence the response of the device to optical radiation.

The measurement of high power and pulsed lasers poses challenging technical problems that have led to the development of specialized detectors, including electrically calibrated ones. We will review the NIST program in laser power and energy measurements and its detector technology in Sec. 5.

The national laboratories check the veracity of their absolute optical power measuring instruments through a continual series of international intercomparisons and other activities designed to establish equivalence of techniques. While a device such as a silicon photodiode can be considered an absolute detector under specified conditions, the actual accomplishment of the task may require considerable expertise and careful attention to specified procedures. Checking the procedures and practices used in the realizations of absolute optical power measurement with a particular detector system is the main impetus for the intercomparisons carried out by the national laboratories.

Due to their importance in modern radiometry and photometry, we will briefly review the use of silicon photodetectors and cryogenic radiometers while leaving discussion of the many other detectors to the literature references cited in this paper.

### 2.1 Silicon Photodiodes

When photons are absorbed by silicon they create pairs of electrons and holes. When this occurs in the junction region of a silicon diode, an electrical current is generated which is proportional to the number of photons absorbed. The energy of the photon must exceed the band-gap energy of silicon, which is about 1.12 eV and which corresponds to light with wavelengths less than about 1200 nm. Silicon’s band-gap allows it to be used as a photodetector from the near infrared into the soft x-ray region [8, 9]. Other semiconductors with lower band-gap energies, such as germanium and indium-gallium arsenide, can be used for detectors further into the IR region.

The responsivity of a silicon photodiode is its output electrical signal per unit of input optical power. The electrical signal is usually the photocurrent measured in amperes and the optical power is measured in watts; hence, responsivity is typically expressed in units of A/W. When this quantity is measured as a function of wavelength, it is referred to as the spectral responsivity of the detector. In most cases the silicon photodiode is coupled to a current-to-voltage amplifier that provides an output voltage that is easily measured with widely available high-quality voltmeters. (For other types of photodetectors and bolometers, the transducer output may be more conveniently expressed in terms of other parameters that match their underlying physics.)

Top quality silicon photodiodes have proven to be generally stable, uniform, and sensitive. In addition, Geist and Zalewski showed that in the visible and near IR wavelength regions the absolute response of certain silicon detectors could be determined by a procedure that they called “self-calibration” [10, 11]. This procedure relies upon the fact that the internal quantum efficiency of silicon is very close to unity over the wavelength region of 500 nm to 950 nm and that the reflectance of the diodes can be accurately measured using ordinary optical techniques. The internal quantum efficiency is the ratio of the number of electron-hole pairs created per absorbed photon. By accounting for the reflected light that is not absorbed, the number of photons incident on the silicon detector can be determined by measuring the electrical current produced. This procedure can, with some care, provide absolute calibrations of silicon detectors used to measure optical power, with a relative standard uncertainty of less than 0.05 % [10].

The reflectance of a silicon photodiode can change as its surface changes, because of additional oxidation, contamination, or even humidity changes in ordinary laboratory atmospheres. Nevertheless, Zalewski and Duda showed how these effects can be minimized by using multiple photodiodes in a “trap” configuration [12]. In a trap configuration, a collimated beam of light reflects off of one photodiode onto another, and then another, until a sufficient number of photodiodes absorb substantially all of the light.

A trap detector is so-named because almost none of the incident light escapes. Most importantly, small changes in the reflectance of an individual surface do not significantly affect the total absorption of the whole device. The light flux not absorbed Φ_na_ is related to the reflectance as shown in Eq. (1):

$$\Phi_{\text{na}}\approx \Phi_{0}\rho^{n},$$ (1)

where *ρ* is the reflectance, *n* is the number of reflections, and Φ_0_ is the incident flux. Since *ρ* is typically about 0.2, Φ_na_ is a small fraction of the incident flux for *n* ≥ 3. Typically, over 99.9 % of the light is absorbed and converted to electrical signal.

Since the internal quantum efficiency is near unity for a substantial wavelength region, trap detectors can be used as absolute standards based upon their known physical properties. In many other cases the inherent stability of trap detectors leads to their use as transfer standards. These are first calibrated against another radiometer, typically a cryogenic radiometer.

**Fig. 2:**
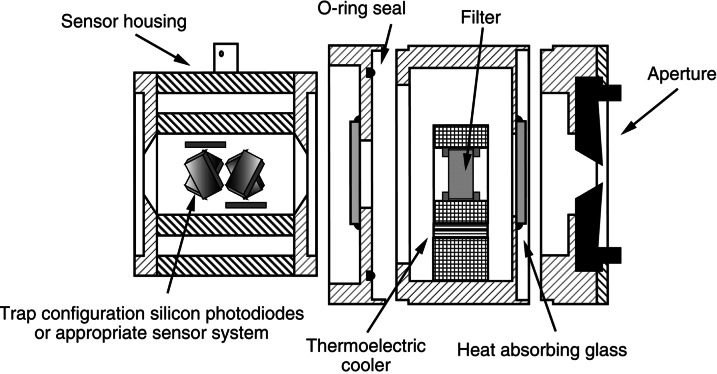
Cross section diagram of a typical photodetector system. Each of the major elements of the system is placed in a separate module for ease of optimizing it for a particular application. The diameter is typically 5 cm to 8 cm and the length varies depending upon the application.

A schematic diagram of a photodetector system used at NIST is shown in Fig. 2. The device is modular and constructed in four stages. This allows us to assemble a detector system including only those stages required for an application.

In many applications a quantity like irradiance or illuminance needs to be measured, and hence a precision aperture is required to define the acceptance area of the detector system. In radiance or luminance measurements it is sometimes necessary to define the configuration factor of a system. This also requires a precision aperture in order to account properly for the flux transferred from one region to another. The design shown in Fig. 2 allows for the attachment of apertures for these purposes.

When a filter is used, as shown in Fig. 2, the system is referred to as a filter radiometer (FR). Filters are important when the application requires that the spectral responsivity of the detector be different than that of native silicon. This is the case in photometry where it is necessary to filter the light such that the response of the photodetector system is closely proportional to the *V*(λ) function. In other applications, narrow band filters may be necessary to radiometrically determine the temperature of a source or to simply monitor a process with optical emission at some particular wavelength. This is often the case in remote sensing applications. For stability it is often necessary to temperature control the filter with thermoelectric coolers or other means to avoid wavelength shifts. In many applications unwanted infrared radiation can be blocked with appropriate absorbing materials. However, in a few applications such as monitoring the output of a monochromator, no filter is used. In this case the spectral responsivity of the detector module is first calibrated and then used to determine the spectral output of the monochromator. Similarly, a calibrated detector can be used for directly measuring the power output from a single frequency laser.

Figure 2 shows a detector system composed of six silicon photodiodes arranged in trap configuration. In this configuration, the collimated light beam entering from the right would make six reflections and be mostly absorbed with only a small residual amount exiting the sensor system after the last reflection [Eq. (1)]. This transmission-trap design allows the untrapped optical flux to be measured, and hence small corrections to the responsivity can be made for extremely accurate measurements.

Instead of a trap detector system, a single silicon detector is often used for simplicity of construction and use. Since filters and other absorbers must be calibrated for transmittance in order to determine the spectral responsivity of the system, when they are used the advantage of a trap detector as an absolute standard is lost. (While one could measure the filter transmittance separately from a well-characterized detector, it is most often expedient to calibrate the entire system together.)

To enhance the stability of the system, the photodetectors can be temperature controlled with a thermoelectric cooler or other suitable temperature-control system. In some applications it is sufficient to use a regulated heater to maintain the detector at a constant temperature above ambient [13].

Silicon photodiodes are available with shunt resistances of greater than 1 GΩ. This large internal resistance allows the construction of high-gain current-to-voltage converters. Signals from silicon photodiodes can have electrical noise floors as low as 0.1 fA [14]. Silicon photodiodes can also have a linear response over many orders of magnitude of input optical power [15]. This stability, linearity, and ease of use have made the silicon photodetector the essential ingredient in contemporary photometry and in many aspects of spectral radiometry. The calibration of filter detector systems and their use will be discussed in later sections of this paper.

### 2.2 Electrical Substitution Radiometry

Electrical substitution radiometers (ESRs) are instruments that measure optical power by equating it to an equivalent amount of electrical power. ESRs are sometimes referred to as electrically calibrated radiometers (ECRs) or simply as absolute radiometers.

**Fig. 3:**
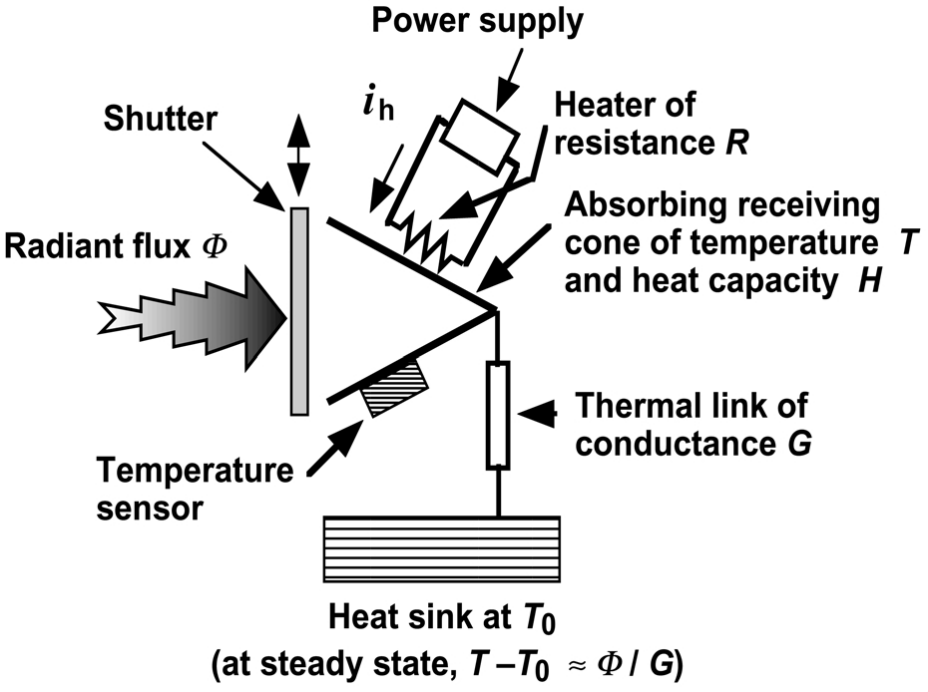
Block diagram of an electrical substitution radiometer (ESR) that shows the major thermal and electrical features. The physical size of the device depends upon the power level of the radiant flux to be measured and can be from fractions of a centimeter to many centimeters in diameter.

The fundamentals of an ESR can be understood by reference to Fig. 3. The radiant flux (optical power) Φ impinges upon a receiving cavity that is designed to collect radiation optimally. Upon absorption of the power, the cavity will experience a temperature rise. The receiver cavity is coupled through a thermal conductor of conductance *G* to a constant temperature heat sink maintained at a reference temperature *T*_0_. Ignoring losses due to radiation, convection, and stray thermal conductance, the equilibrium (long-time) temperature rise is given by *T T*_0_ = Φ/*G*. The same amount of power supplied by the electrical heater would have the same effect.

In practice, ESR performance can often be improved by maintaining the receiving cavity at a constant temperature with the electrical heater. When a shutter is imposed to stop the light beam, power is supplied to the heater to create a set temperature rise. When the shutter is opened, a feedback loop decreases the electrical power a sufficient amount to maintain the same cavity temperature. Ignoring corrections and losses mentioned earlier, the optical power is given by Φ= (*i_c1_*^2^− *i*_op_^2^) *R*, where *i*_cl_ is the current applied through the heater of resistance *R* when the shutter is closed and *i*_op_ is the current when the shutter is opened.

While we have used a cavity absorber for this example, the idea of electrical substitution is used to calibrate other optical detectors such as bolometers and pyroelectric devices. These are often used for laser power and energy measurements (discussed in Sec. 5). The practical challenge when utilizing ESRs is to carefully characterize the various loss mechanisms in order to apply appropriate corrections to the power equivalence relationship.

ESRs have been in use for 75 years or more, and their history and development have been described by Hengstberger in considerable detail [16]. ESR technology was pioneered by Coblentz at NBS during the early part of this century [17]. He developed a number of radiometers and used them for diverse purposes in photometry and radiometry, including an early measurement of the Stefan-Boltzmann constant [18]. The limited wavelength range and difficulty in obtaining consistently high accuracy with solid-state devices spurred the development of much-improved ESRs during the 1970s and 1980s.

In this time period, a significant innovation was the design and construction of an ESR that operated at cryogenic temperatures. This resulted in an increased sensitivity and a reduction in the uncertainties due to radiative and convective losses. The first cryogenic radiometer at NBS was constructed by Ginnings and Reilly in 1972 to measure thermodynamic temperatures above 0 °C [19]. For a variety of reasons this project did not achieve the desired results, but by building on the experience gained by Ginnings and Reilly, Yokley built a cryogenic radiometer system at NBS in the mid-1970s to measure the radiation temperature of low-temperature blackbodies used in a low-background environment [20, 21]. This device was used for a number of years to perform specialized calibrations of low-flux sources, but it was not engineered to perform high-accuracy measurements. It yielded measurements with relative uncertainties of several percent.

Quinn and Martin at the National Physical Laboratory (NPL) in the UK developed a high-accuracy cryogenic radiometer for use in a radiometric determination of the Stefan-Boltzmann constant with a relative combined standard uncertainty of 0.013 % [22]. In order to achieve this low uncertainty, it was necessary to measure radiometric flux with this same uncertainty. This important benchmark work contains a detailed analysis of the errors and uncertainties associated with a cryogenic ESR and has led to the adoption of these devices as fundamental radiometric standards with relative combined standard uncertainties of less than 0.01 %. NPL staff later developed this device into a radiometer for laser power measurements and thereby provided a technique to establish high accuracy calibrations of other stable detectors by comparison to the cryogenic radiometer [23]. The commercial availability and intrinsic accuracy of these instruments has resulted in their employment by a number of national standards laboratories to provide the technical basis of radiometric measurement [24].

**Fig. 4:**
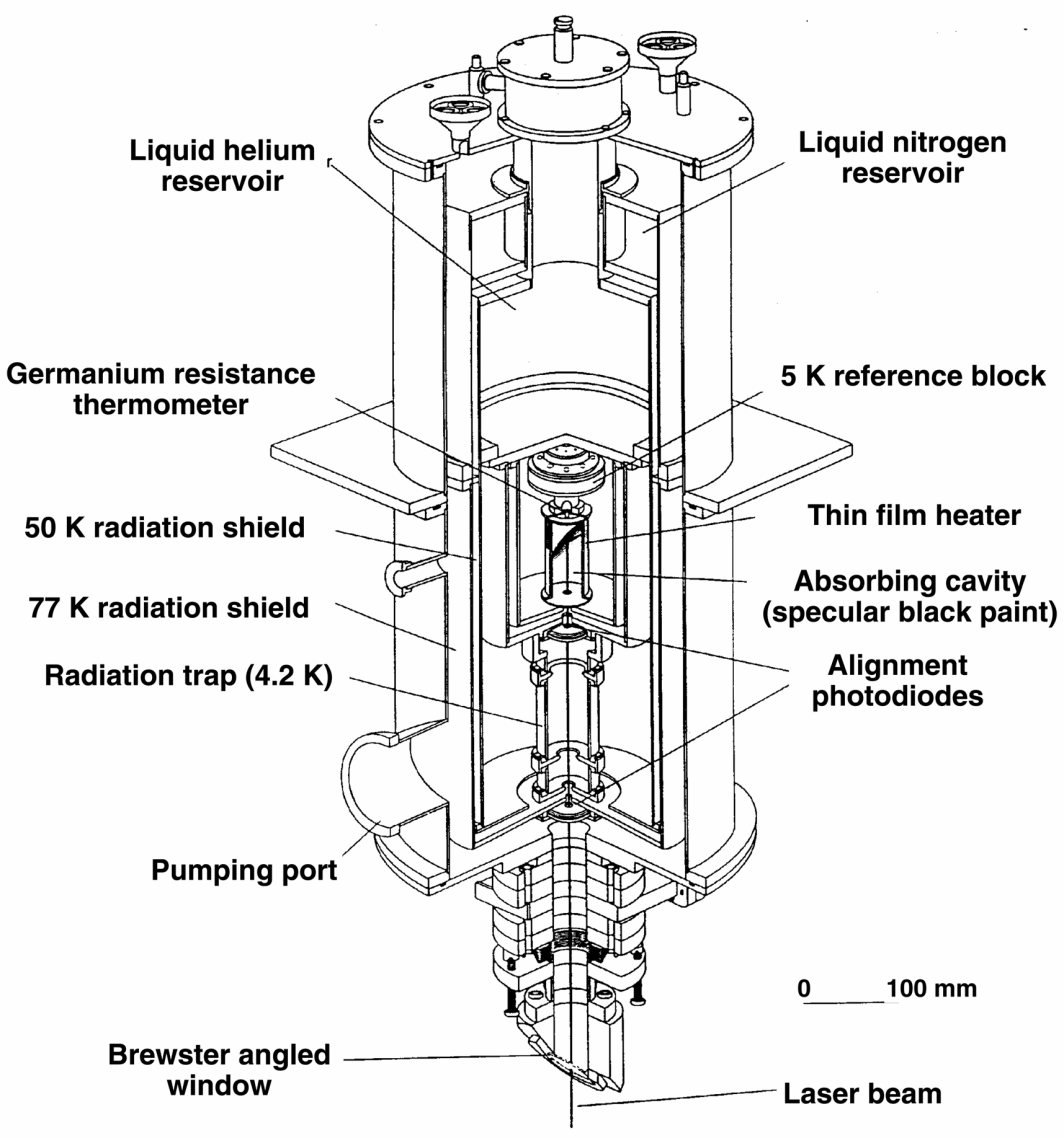
Breakaway diagram of High-Accuracy Cryogenic Radiometer (HACR). The HACR is a primary standard for optical power measurement.

**Fig. 5:**
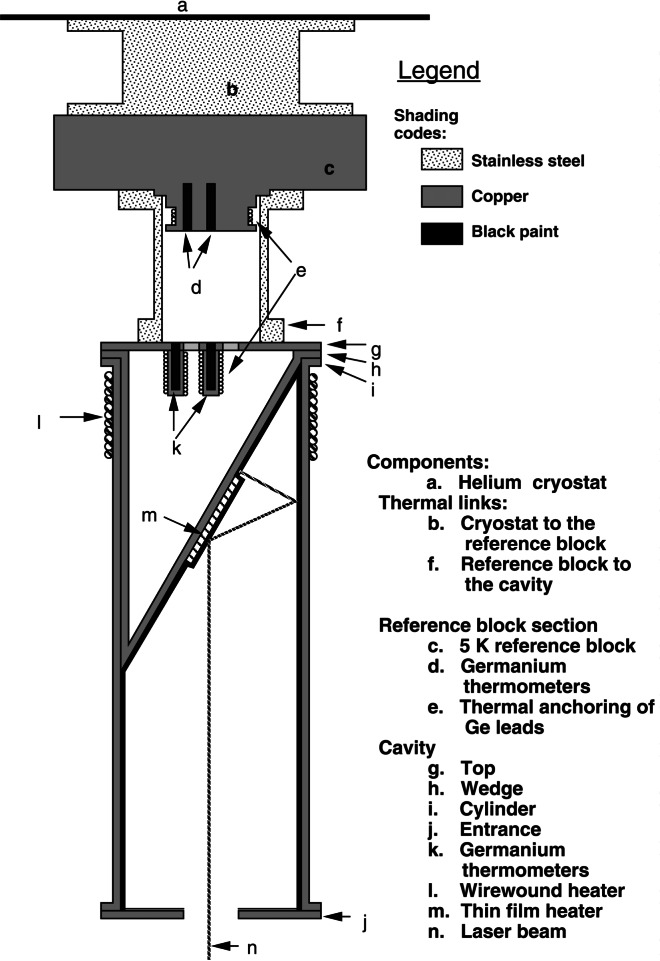
Detailed cross section drawing of the High-Accuracy Cryogenic Radiometer’s absorption cavity and associated thermal and electrical elements.

The NIST High-Accuracy Cryogenic Radiometer (HACR) is shown in Fig. 4 and is described in detail in the technical literature [25]. The radiometer system is contained in a large stainless steel vacuum vessel that contains reservoirs for cryogenic fluids. The outer cryogenic shield is maintained at 77 K with liquid nitrogen, and the inner reservoir is filled with liquid helium at 4.2 K. An intermediate shield stabilizes at an operating temperature of about 50 K. The heart of the instrument is the absorbing cavity that is connected to a thermally controlled heat sink held at 5 K. An enlargement of the cavity is shown in Fig. 5. Polarized optical radiation from a laser system enters the vacuum vessel of the HACR through a window at Brewster’s angle. After passing through the window the light is incident upon the absorbing cavity, which is designed so that the optical radiation undergoes many reflections and is effectively trapped in the cavity. The cavity is coated with a specular black paint to improve the absorptance. The substantial distance the light must travel in the HACR before being absorbed requires that the light be highly collimated. This is most often accomplished by using laser radiation. Laser radiation also provides higher power levels, typically milliwatts of power, than are commonly available with a monochromator system. This level of power is required by the first generation of cryogenic radiometers. Newer systems are now available which can be used successfully with radiation from a monochromator and conventional light sources.

The reference block, (c) in Fig. 5, is actively maintained at a constant temperature and acts as a heat sink for the cavity. The temperature of the present HACR reference block is maintained to within 0.15 mK over a typical measurement time of 45 min. The temperature is sensed by germanium resistance thermometers, (d) in the figure. There is a thermal link (b) from the reference block to the helium cryostat (a) and a second one (f) from the cavity to the reference block. There are two different heater arrangements on the cavity, one of them (m) is at the approximate position where the laser beam is incident and the second (l) is wrapped around the outer portion of the cavity. Both can be used for the electrical substitution equivalence measurement and their separate positions allow checking the effectiveness of the heating system for equivalence of optical and electrical power absorptance by the cavity. This instrument achieves a measurement of optical power to within a 0.021 % relative combined standard uncertainty [25, 26] for optical powers on the order of 1 mW. Because of this impressive accuracy, the HACR is a national standard for many optical power measurements in the visible wavelength region.

**Fig. 6:**
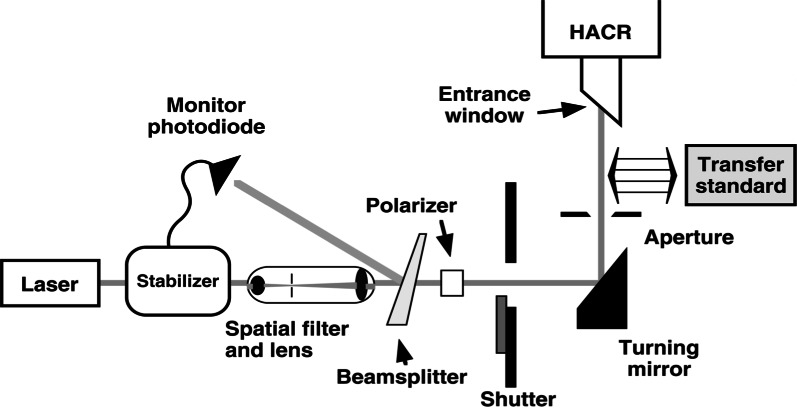
Schematic representation of the methodology of using the High-Accuracy Cryogenic Radiometer to calibrate transfer standard detectors.

A typical mode of operation for the HACR is shown schematically in Fig. 6 and is described in more detail in the literature [25, 27]. A secondary or transfer standard detector (TSD) is inserted into the laser beam and intercepts the same beam that is measured by the HACR. After corrections for the HACR entrance window transmittance and other systematic effects, the known optical power of the beam is used in the computation of the responsivity of the TSD. (A TSD is used to calibrate other instruments and devices, as discussed in Sec. 2.3.)

A complete calibration for a TSD consists of determining the absolute spectral responsivity (ASR) of the device, using both the HACR measurements and appropriate modeling. Here, the term “absolute” is used to indicate that the spectral responsivity scale is derived from the national standards. This distinguishes these measurements from the more common situation where a relative responsivity function is shown to merely compare the performance of a detector at different wavelengths.

The ASR of a TSD can be calibrated directly using the HACR at selected laser wavelengths, and then the responsivity for regions between the laser wavelengths can be interpolated based upon knowledge of the physics of the TSD. In the case of silicon photodiodes, a considerable amount of effort has gone into modeling their responsivity as a function of wavelength and hence the spectral responsivity from 400 nm to 1000 nm can be accurately deduced [11, 28]. Gentile and co-workers used this technique to develop the NIST scale of detector spectral responsivity with a relative combined standard uncertainty of less than 0.04 % in the wavelength range of 406 nm to 920 nm.

Other types of detectors can be calibrated using this substitution technique in different wavelength regions, including spectrally flat absorptive bolometer detectors and other semiconductor devices [7]. A bolometer can be utilized as a TSD by carefully characterizing its stability and spectral absorption properties. The bolometer can be calibrated at selected wavelengths with the HACR and its overall spectral response characteristics inferred from knowledge of its relative spectral absorptance.

In some wavelength regions lasers may not be readily available for use in the manner shown in Fig. 6. So, cryogenic radiometers have been developed that operate with monochromatized light provided by conventional optical sources, which produce a continuous wavelength coverage for calibration [29]. These monochromator-based sources generally have a more diverging beam and less optical power per wavelength interval than do lasers. Their use with a cryogenic radiometer to characterize a TSD can, therefore, require greater care to achieve equivalent levels of uncertainty. Recently, cryogenic radiometers have been installed at synchrotron radiation facilities. With appropriate monochromators they afford accurate measurement over a wavelength region from the far UV to the IR spectral regions [30, 31].

The use of a cryogenic radiometer usually relies upon the availability of suitable window materials, which presents technical challenges in some wavelength regions. However, in a vacuum environment, such as with a synchrotron source, the necessity of using a window is avoided. While window corrections no longer contribute to the overall measurement uncertainty, other problems associated with ensuring that a particular TSD operates equivalently in a vacuum and in the air can pose a difficulty.

### 2.3 Detector Spectral Responsivity Measurements

The ultimate goal of detector-based radiometry is to determine the spectral responsivity of any photodetector system in terms of the spectral responsivity of an absolute national standard, its ASR. One approach might be to calibrate many detectors against a cryogenic radiometer standard directly, presuming that both the cryogenic radiometer and the detectors under test could utilize the same light source. However it is usually preferable and more expedient, because of the complexity of a cryogenic radiometer, to make the comparison in two steps—first from the cryogenic radiometer to a TSD, and then from the TSD to the detector of interest. While the accuracy of a TSD-based calibration may be somewhat diminished, the ease of use and the possible avoidance of maintaining a cryogenic system make this approach useful in applications where the utmost calibration accuracy is not required. One also gains latitude to use a light source more appropriate for the absolute standard in the first step (e.g., a fixed-wavelength laser), and another source more appropriate for the detector under test in the second step (e.g., a tunable monochromator).

For these reasons, and to provide calibrations for a wide range of customers and uses, NIST maintains a set of instruments that utilize monochromators and which are known as detector spectral comparators (DSCs) [32, 33]. NIST has DSCs engineered for the UV, visible, near IR, and far IR spectral regions in addition to a facility at the NIST synchrotron that also can provide calibrations over a broad wavelength region.

**Fig. 7:**
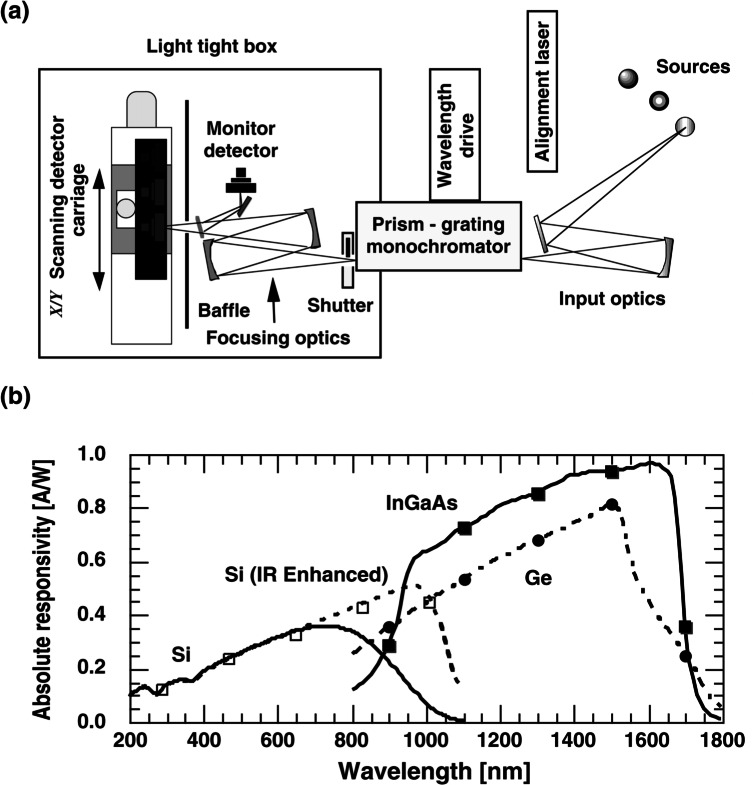
a) Layout diagram of the UV-visible Detector Spectral Comparitor (DSC). b) Typical results of calibrating solid-state detectors on the DSC. The horizontal axis is wavelength in nanometers (nm) and the vertical axis is the absolute spectral responsivity in A/W.

Figure 7a shows a schematic outline of the NIST visible-near-IR DSC measurement facility. The essential features are a set of light sources chosen to cover the various wavelength regions, input optics, a double monochromator, and exit optics to refocus the light on the detectors being calibrated or on a TSD being used as the calibration standard. In practice the TSD is calibrated using the HACR, mounted on a moveable carriage, and then placed in position to receive light from the monochromator. Its signal is determined at a particular wavelength, and then the test detector is moved to the same position. A monitor system ensures the stability of the monochromator output during the two measurements. Using this procedure, the ASR of the test detector is established in terms of the TSD, and hence in terms of the HACR. Typical results are shown in Fig. 7b.

Detector systems often have aperture areas of about 1 cm^2^ or greater, and the ASR measurements are usually performed by using the focused beam from the monochromator, usually a few mm^2^. Hence, the entrance aperture of a detector is often underfilled. However, the carriage provides vertical and horizontal motion normal to the exit beam of the monochromator and allows for the mapping of the spatial uniformity of the detector’s ASR.

**Fig. 8:**
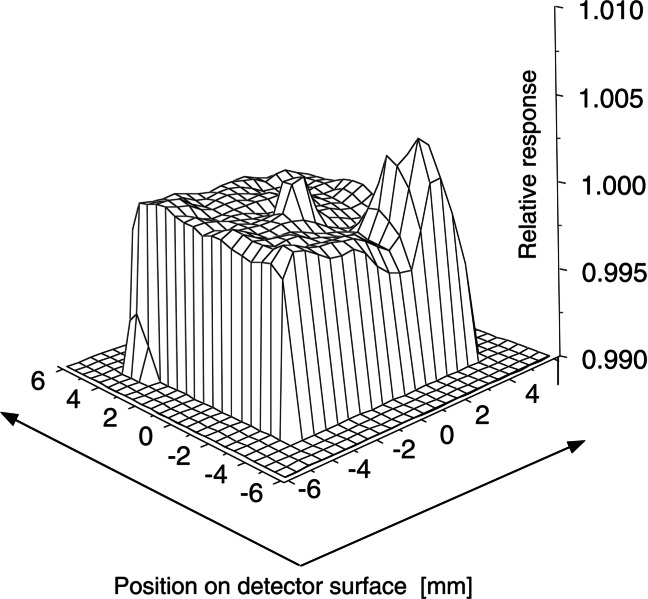
Typical results of characterizing the spatial uniformity of the spectral responsivity of a solid state detector. The vertical scale is the relative response normalize to the value at the center of the device and the horizontal scales are locations on the detector surface measured in mm.

Figure 8. shows typical spatial uniformity data for a silicon detector where the uniformity of response is within 1 % over the entire active area. This uniformity is usually a function of wavelength and hence a full characterization of a detector will include a study of the uniformity over the wavelength regions where it might be used. These measurements are important as not all detectors are this uniform and, depending on the active area used in a particular application, an appropriate average responsivity must be calculated.

This averaging is necessary to determine the wideaperture response for irradiance or illuminance measurements where the detector aperture is overfilled. Examples of this type of measurement are the determination of the illuminance from a lamp or the spectral irradiance of a source like the Sun. However, a lack of uniformity and the averaging process give rise to increased uncertainties in the ASR of detectors used in these applications.

Instead of calibrating the instrument by determining the responsivities of the different regions of the active area, and averaging them, a better strategy is to calibrate the instrument with a uniform field of known illuminance or irradiance. To improve the calibration capability for these types of applications and to avoid the averaging method mentioned above, NIST has developed a new laboratory, the Spectral Irradiance and Radiance Calibration using Uniform Sources (SIRCUS) facility, for these types of calibration [34].

**Fig. 9:**
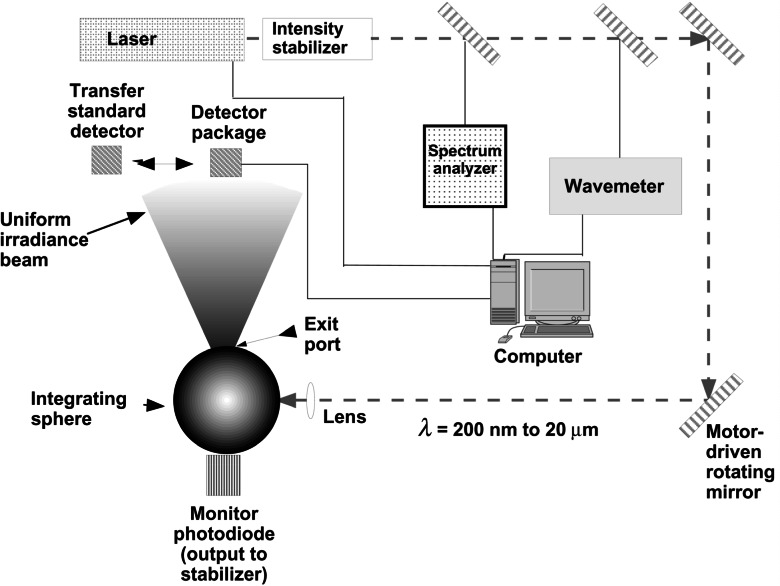
Schematic layout of the Spectral Irradiance and Radiance Calibration using Uniform Sources (SIRCUS) facility used to calibrate irradiance spectral responsivity of filter radiometers and other optical sensor systems. SIRCUS has lasers that provide radiation from 200 nm to about 20 m. The major system components are labeled in the figure itself.

Figure 9 gives a schematic diagram of the SIRCUS facility. It has as a core feature a suite of power-stabilized laser systems covering the wavelength region from 200 nm to beyond 20 μm. The integrating sphere provides a spatially averaged beam that is uniform in intensity over a detector aperture. In this arrangement a TSD that has been carefully characterized for ASR and spatial uniformity, and fitted with a precision aperture, is used to determine the irradiance where test detectors are later substituted in position. Using this procedure, the irradiance responsivity of a test detector system can be determined in a manner that is insensitive to the details of the test detector aperture, provided that the test detector intercepts approximately the same portion of the calibration beam that the TSD utilized.

An advantage of this facility is that by knowing the aperture areas of the detectors and the aperture area of the integrating sphere exit port, SIRCUS can be used as a radiance source to calibrate sensor systems that are designed to measure spectral radiance. A good example comes from pyrometry, where the determination of the spectral radiance of a blackbody source can be used to infer its temperature using the Planck law. In order to provide precision apertures for these purposes, NIST has developed a new facility to accurately determine aperture areas so that their uncertainties will not limit the accuracy of radiometric and photometric measurements [35].

The array of facilities briefly described here allows NIST to establish scales for the radiometric and photometric SI quantities shown in Table 1 with measurement chains linking back to the HACR. As the technology for using cryogenic radiometers progresses, we will implement these changes and thereby enhance our photometric and radiometric measurement standards for NIST’s customers. This technical approach has been embraced worldwide. Most national laboratories engaged in maintaining their own radiometric and photometric units have established, or are establishing, a cryogenic-radiometer-based system of measurement [24].

## 3. Photometry

The SI base units for mass, length, time, temperature, electrical current, and the quantity of matter are completely described in terms of physical properties, but the base unit for luminous intensity is narrowly defined by optical power measurement at a single wavelength. However, within SI, the candela stands apart from the other six base units in that it is anthropocentric. The purpose of photometry is to measure all light with a metric related to human vision, so one extends the definition of the candela to other wavelengths using *V*(λ) [5]. This aspect of its definition also suggests methods for the realization of the unit.

### 3.1 Candela

The photometric quantities in Table 1 are related to the spectrally-dependent radiometric quantities by integrating them over the wavelengths where the eye is sensitive, as weighted by *V*(λ).^1^ For example, the luminous flux is related to the radiant flux:

$$\Phi_{v}=K_{m}\int_{\lambda }\Phi \left(\lambda \right)V\left(\lambda \right)d\lambda ,$$ (2)

where *K*_m_ is the maximum spectral efficacy of radiation for photopic vision. As a result of the 1979 definition of the candela, *K*_m_ is equal to 683 lm/W [36]^2^. The integral in Eq. (2) is evaluated over the range of wavelengths where *V*(λ) is non-zero, which as a practical matter is a wavelength range from 380 nm to 780 nm. This equation formally expresses the means of providing photometric information about light sources or visual illumination systems, such as building lighting, by integrating the appropriate radiometric quantities with a *V*(λ) weighting.

**Fig. 10:**
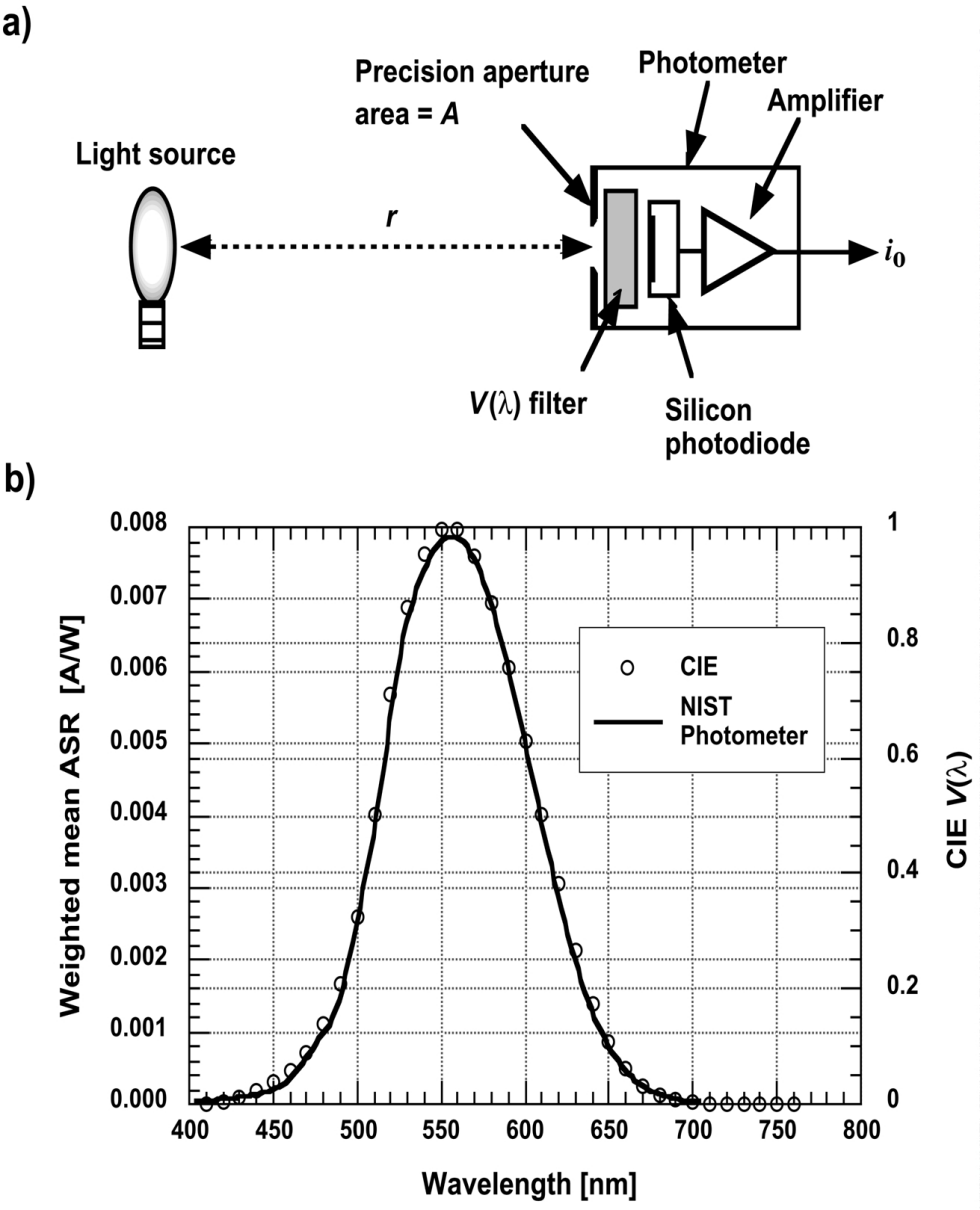
a) Schematic diagram of how the candela is maintained using a photometer. The major features of the photometer and the details of its use are labeled in the figure. b) The average absolute spectral response of a NIST photometer used for maintenance of the candela and other photometric quantities. The left vertical axis is the absolute spectral response of the photodetector in A/W and the right vertical axis is the CIE luminous efficiency distribution, which is dimensionless and normalized to unity at 555 nm. The horizontal axis is wavelength in nm.

This integration can be done physically by constructing a photodetector system with a spectral responsivity proportional to the *V*(λ) function. That is, one need not make spectrally resolved measurements followed by a numerical integration. This is shown schematically in Fig. 10a. A light source with some spectral distribution is placed at a distance *r* from a photodetector. The system is designed such that combined filter and detector give an overall responsivity proportional to the *V*(λ) distribution. Therefore, the output current *i*_o_ is composed of signal from all the spectral components of the light, each receiving the appropriate weighting.

Figure 10b shows the data for the ASR calibration of one of NIST’s photometers that is used for routine photometric measurements. Also plotted for reference is the *V*(λ) distribution where the vertical axes have been normalized for ease of comparison. In this instance, as can be seen from the figure, the combination of the filter and detector responsivity produced an ASR that matches the shape of the *V*(λ) function very closely. The small differences between *V*(λ) and the ASR for a photometer can be accounted for, and small corrections to a measurement result can be made [4, 37].

To understand how the photometers can be used to establish the photometric quantities, consider the arrangement of the lamp in Fig. 10a that produces a spectral irradiance *E*(λ) on the plane of the detector whose aperture has area *A*. The area of the aperture is assumed to be small with respect to the distance *r*, hence the irradiance is assumed to be uniform over the aperture. (In an actual experiment or calibration these assumptions would be verified and an uncertainty assigned.) The result is that the spectral radiant flux is given by *A E*(λ). If the ASR of the photodetector is *s*(λ) the output current *i*_o_ of the detector system will be given by,

$$i_{o}=A\int_{\lambda }E\left(\lambda \right)s\left(\lambda \right)d\lambda$$ (3)

following directly from the definition of the ASR. The gain of the amplifier is ignored in this discussion and the output will be taken as a current even though in practice a current-to-voltage converter may be used. The transfer properties of the amplifier can be very accurately determined, but this is beyond the scope of the present discussion.

Alternatively we can express the output current in terms of a luminous flux responsivity *s*_v,f_ expressed in the unit A/lm and defined in the following manner:

$$s_{v,f}=\frac{i_{o}}{\Phi_{v}}=\frac{i_{o}}{A\;K_{m}\int_{\lambda }E\left(\lambda \right)V\left(\lambda \right)d\lambda }.$$ (4)

This follows directly from Eq. (2), presuming that the irradiance is constant over the aperture area A. Combining Eqs. (3) and (4) gives:

$$\begin{array}{l} s_{v,f}=\frac{\int_{\lambda }E\left(\lambda \right)s\left(\lambda \right)d\lambda }{K_{m}\int_{\lambda }E\left(\lambda \right)V\left(\lambda \right)d\lambda }\\ =\frac{s(555\text{nm})\int_{\lambda }E\left(\lambda \right)s_{n}\left(\lambda \right)d\lambda }{K_{m}\int_{\lambda }E\left(\lambda \right)V\left(\lambda \right)d\lambda }. \end{array}$$ (5)

For convenience and to make our connection to the definition of the candela more apparent, we have factored *s*(λ) into *s*(555 nm) *s*_n_(λ). The term *s*(555 nm) is the absolute spectral responsivity at 555 nm (the peak position of the *V*(λ) function). The term *s*_n_(λ) is the relative spectral responsivity, normalized to unity at 555 nm (also the value of *V*(λ) at its peak).

Clearly, if *s*_n_(λ) were to equal *V*(λ) then the two integrals would cancel. In practice, with modern technology and optical fabrication skill, one can achieve a reasonably close match. This allows for the simplification

$$s_{V,f}=\frac{s\left(555\text{nm}\right)}{K_{m}}(1+\text{correction}\;\text{terms}),$$ (6)

where the correction terms depend upon the spectral mismatch between *s*_n_(λ)) and *V*(λ)) as well as the spectral distribution of the light.

A filtered detector system designed in this way, with a spectral responsivity that mimics *V*(λ), is called a photometer. Its luminous flux responsivity can be determined by knowing the ASR at a wavelength of 555 nm, and constants.

In the same way that we wrote the spectral radiant flux received by the photometer as *A E*(λ), we can write the luminous flux as Φ_v_ = *A E*_v_, where *E*_v_ is the illuminance expressed in lux [lx]. (1 lx = 1 lm/m^2^.) It follows that the illuminance responsivity *s*_v,i_ of a photometer is:

$$S_{V,i}=As_{v,f}.$$ (7)

For the case of a point-like light source, which is realized by having the dimensions of a lamp and the photo meter aperture small compared to *r*, the distance between them, the relationship between the illuminance *E*_v_ and luminous intensity *I*_v_ is:

$$E_{v}=\frac{I_{v}}{r^{2}}=\frac{i_{o}}{S_{v,i}}=\frac{i_{o}}{A\;S_{v,f}}.$$ (8)

Solving for *I*_v_ we find,

$$I_{v}=\frac{i_{o}r^{2}}{S_{v,i}}=\frac{i_{o}r^{2}}{As_{v,f}}.$$ (9)

Luminous intensity is thereby determined from the signal from a calibrated photometer and geometrical factors. Most importantly, the 1979 redefinition of the candela allows this to be an approach for the realization of a luminous intensity scale—quite a simplification from the previous requirement to use a platinum-point blackbody source. This procedure has the additional attraction that uncertainty in a candela scale can be reduced as the ability to measure the illuminance responsivity of a photometer improves.

The illuminance responsivity, as can be seen from Eqs. (6) and (7), depends directly upon the ASR value at 555 nm, aperture area, a known constant (*K*_m_), and correction terms. Today, the stability and continuity of the SI unit relies upon the ability to construct and maintain photometers whose ASR is known with low uncertainty. The advent of cryogenic radiometer technology and the availability of stable silicon detectors have considerably advanced the ability to measure ASR, but this factor still is a limiting factor in improving the measurement of luminous intensity.

At NIST, the calibration of *s*_v,f_ (or *s*_v,i_) is traced to the HACR. The current realization of the NIST candela has a relative expanded uncertainty of 0.41 % (coverage factor *k* = 2) with the major component of the uncertainty due to the uncertainty in the illuminance responsivity [38, 39]. This is roughly a factor-of-ten increase in uncertainty over the uncertainty of a HACR measurement at the beginning of the measurement chain. The additional uncertainty accumulates through the TSD comparison steps and arises due to geometric issues in the transition from flux measurement to irradiance measurement. The development of SIRCUS is expected to improve the measurement of ASR and irradiance by a factor of five or so and thereby improve the photometric scales by a similar amount [34]. Our initial effort using SIRCUS to calibrate filtered detectors for radiation temperature measurements indicates that this expectation is realistic and that we can safely predict ongoing improvement in measurement of photometric quantities [40].

**Fig. 11:**
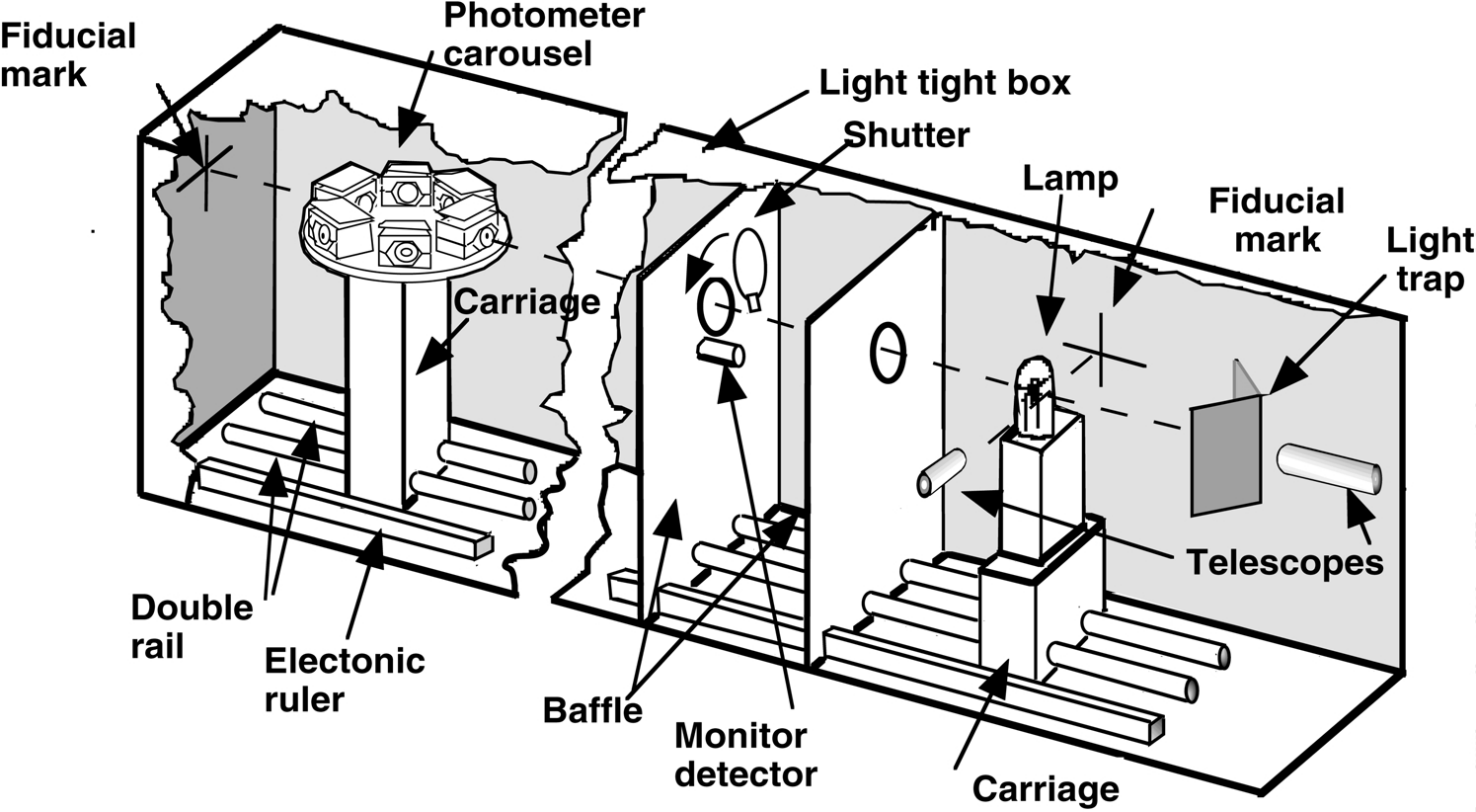

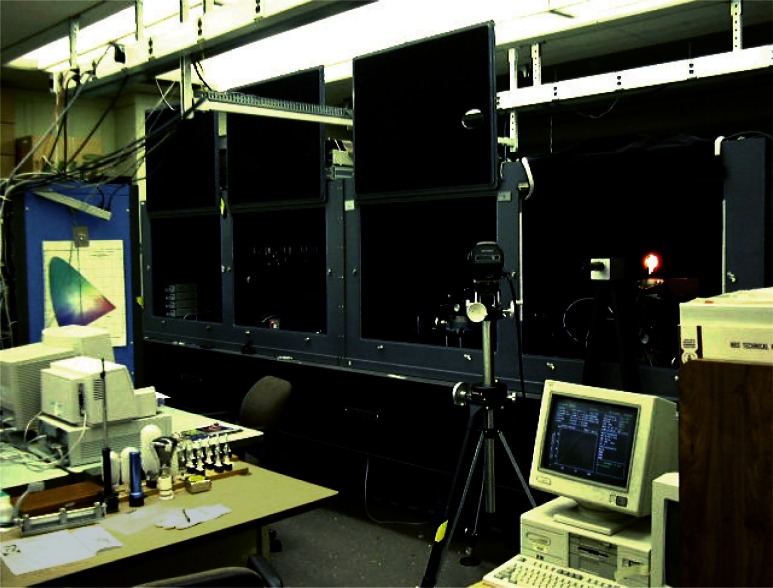
a) Drawing of the NIST photometric bench, which is used for measurement of the luminous intensity of lamps and related photometric quantities. The major features of the instrument are labeled in the drawing. A lamp is placed on the carriage and aligned with the telescopes, and the photometric signal is measured with one of several photometers placed on the carousel at the left side of the drawing.

Figure 11a shows a schematic drawing of the major features of the photometric bench that is used to maintain the NIST candela scale. This bench provides the ability to calibrate customers’ photometers and lamps with respect to the NIST standards. Six photometers are mounted on a carousel that allows the rotation of any one of them into the light beam. The instrument has two alignment telescopes to correctly position the lamp on the optical axis. The distance between the lamp and the photometer entrance aperture is measured with a digital linear encoder that has been calibrated traceable to NIST length standards. The stability of the lamp is monitored with a stable monitor detector mounted on the baffle, and a shutter allows for the determination of the dark currents of the photometers. Dimensional stability is ensured by mounting the entire apparatus on commercially available optical tables. Figure 11b is a photograph of the instrument and ancillary equipment.

### 3.2 Lumen

While the candela is the SI base unit for photometry, the lumen, the unit of luminous flux, is generally of greater commercial interest. A key metric for lamps and illumination devices is their total luminous flux, which is their luminous intensity integrated over the entire solid angle, usually 4π, into which they radiate.

This measurement is usually accomplished using an instrument called a goniophotometer. Goniophotometers are usually large, room-sized devices that provide movement of a photometer over a solid angle of 4π and thereby provide the total luminous flux from direct integration of the luminous intensity (or illuminance) [2–4, 41]. Until very recently this had been the primary method of directly determining the total luminous flux of a light source.

However, Y. Ohno at NIST has developed a new method of accurately integrating the total flux from a lamp, which uses an accurately characterized integrating sphere [39, 42–44]. An integrating sphere is an instrument constructed by coating the inner surface of a spherical shell with a highly reflective and diffusive material. The result is that the light from a lamp inside the sphere undergoes many reflections, which averages the spatial distribution of the lamp and results in a uniform average illuminance at the sphere’s wall. (The integrating sphere concept was introduced to light measurement in 1892 by Sumpner in the course of his work on studying the reflection properties of surfaces and how the reflectances of walls affected the overall illumination in a room [45].)

It can be shown that if a source of total luminous flux Φ_v_ is placed in the interior of a sphere of radius *r* whose reflectance is *ρ*, then the illuminance *E*_v_ at the surface of the sphere is given by Eq. (10) [3]:

$$E_{v}=\frac{\Phi_{v}\rho }{4\pi r^{2}\left(1-\rho \right)}.$$ (10)

The reflectance of a sphere can vary temporally, spatially, and with wavelength, and hence the integrating sphere has traditionally been employed only in the substitution mode for measurement with respect to a standard lamp whose total luminous flux is known by use of a goniophotometer. In the substitution method, a test lamp is compared with a standard lamp of known luminous flux and which has similar distribution of radiation.

**Fig. 12:**
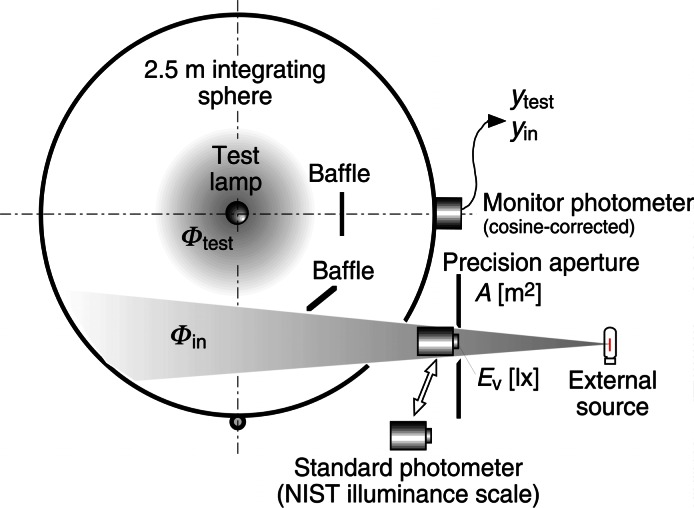

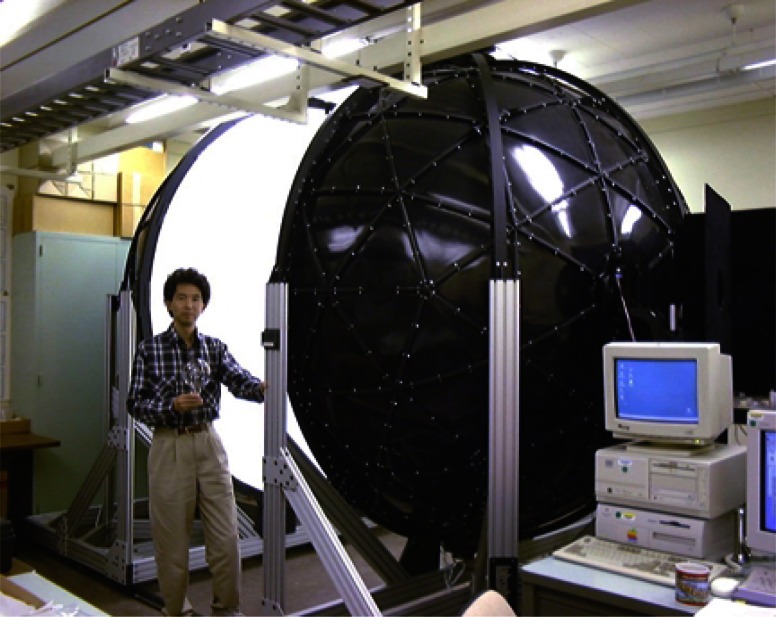
a) Schematic drawing of the NIST 2.5 m diameter integrating sphere used for measuring luminous flux from lamps. Light from the external source is admitted to the sphere through a well-characterized aperture. By knowing the illuminance of the light and the area of the aperture the flux admitted to the sphere is known and can be used to calibrate the flux from the test lamp enclosed within the sphere. b) Photograph of the 2.5 m diameter integrating sphere that shows the details of the construction.

The implementation of Ohno’s method at NIST is shown in Fig. 12a, where a 2.5 m diameter integrating sphere is fitted with a cosine-corrected photometer for use as a monitor for purposes of the calibration. The integration sphere has a hole, shown in the lower right portion in the figure, to allow for the admission of a known external flux Φ_in_. The monitor photometer signal is determined with the externally applied input flux. The external flux is shut off and the monitor photometer then determines the signal due to the test lamp whose flux is shown as Φ_test_. The signals at the monitor photometer are *y*_in_ and *y*_test_, respectively. The external flux, Φ_in_ supplied to the sphere is given by *A E*_v_, where *E*_v_ is the illuminance measured at the precision aperture *A* using a calibrated photometer whose ASR calibration was discussed in the previous section. The test lamp flux is then given by

$$\Phi_{\text{test}}=c\Phi_{\text{in}}\frac{Y_{\text{test}}}{Y_{\text{in}}}.$$ (11)

Here, *c* is a correction factor that contains terms to account for the spatial nonuniformity of the sphere, the oblique angle of incidence of the external source radiation, and the spectral mismatch between the test lamp and the external source. This latter term arises because the combination of the integrating sphere and photometer can have a relative spectral responsivity that deviates from *V*(λ). These combined corrections are on the order of 1 % to 2 % and are known with some certainty; they are not limiting factors in the accuracy of the measurement. Determination of the correction factor involves a detailed angular mapping of the sphere’s response as well as ascertaining its spectral characteristics. These procedures are extensively described in the literature [42–44].

Figure 12b is a photograph of the NIST 2.5 m diameter luminous flux integrating sphere and some of the associated laboratory instrumentation. The sphere is built from preformed plastic sections, and its inside is coated with a fluorocarbon composite material.

Measurement of luminous flux with Ohno’s method has a relative expanded uncertainty of 0.53 % (coverage factor *k* = 2). The primary contribution to the uncertainty arises from the uncertainty in the ASR measurement of the standard photometer. As with the candela, the improvements that are envisioned in ASR measurements utilizing SIRCUS and other new facilities will have a direct impact upon this important photometric quantity.

### 3.3 Illuminance and Luminance

**Fig. 13:**
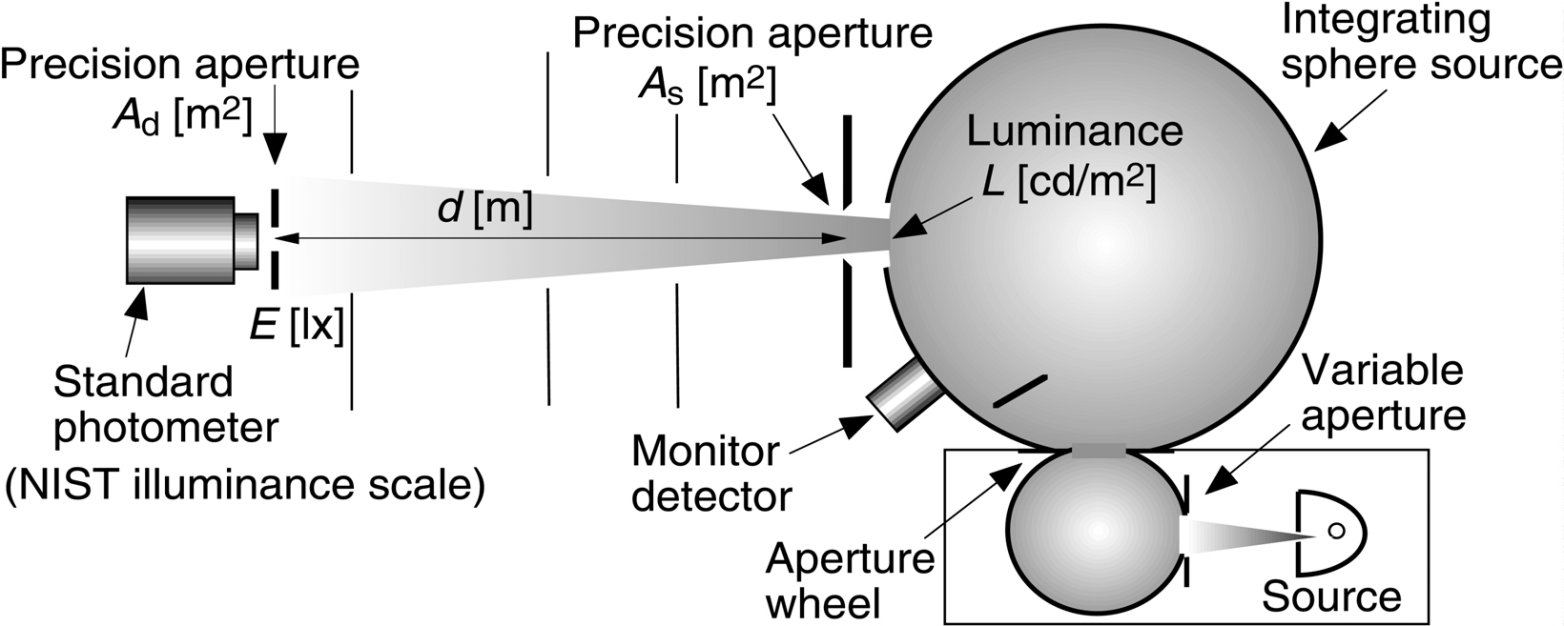
Diagram of the essential features of the instrument used to provide luminance calibrations.

Illuminance and luminance, quantities used extensively in lighting engineering, are related to the spectroradiometric quantities of irradiance and radiance by Eq. (2). Irradiance is a measure of optical power falling on a reference surface, and radiance quantifies light emission, per area and solid angle, by a source. We discussed above how the illuminance response (in A/lx) of a photometer can be determined. Figure 13 shows how this type of photometer can be used to characterize the luminance of a source that is constructed using two integrating spheres. The two integrating spheres are used to improve the uniformity of the light output as well as to allow the introduction of an aperture wheel between them, which provides the ability to change the luminance level over a large dynamic range. The source and sphere configuration produces a uniform illumination at the plane of the sphere aperture *A*_s_. A standard photometer with a precision aperture area *A*_d_ is placed at a distance *d* from the sphere aperture. The luminance of the sphere source *L*_v_ can be related to the illuminance *E*_v_ measured by the photometer [39]:

$$L_{v}=\frac{k\;E_{v}d^{2}}{A_{s}},$$ (12)

where,

$$K\approx \left(1+\frac{A_{s}}{\pi d^{2}}+\frac{A_{d}}{\pi d^{2}}\right).$$

Here, *k* is the configuration factor that accounts for the finite sizes of the apertures. Configuration factors have been tabulated in the literature for most illumination geometries ordinarily used [46, 47].

The luminance of the sphere source can be determined using a calibrated photometer whose illuminance response is known, and then another test instrument that is designed to measure luminance can be calibrated from it. In this manner luminance meters can be directly calibrated for NIST customers using a method based upon the NIST illuminance response determination described earlier in this paper. The monitor detector ensures temporal stability of the system for the transfer measurement and allows for the changing of the sphere-connecting aperture in a controlled way. The linearity of the silicon detectors used in the photometer allows for devices (such as shown in Fig. 13) to be operated over many orders of magnitude of signal output with the same detector configuration. This instrument is used to calibrate luminance meters for NIST customers with respect to the NIST maintenance of luminance that has a relative expanded uncertainty of 0.50 % (coverage factor *k* = 2). The largest source of uncertainty is in the illuminance responsivity of the photodetector and hence this quantity will attain lower uncertainty as the ASR measurements for detectors are improved. The actual calibration uncertainties for the customers’ units may be slightly larger due to vagaries of the supplied commercial instruments.

The detector-based techniques described here for photometry have evolved to the point that NIST now recommends that its customers begin maintaining their photometric and radiometric standards on a detector basis whenever possible [48]. Once adopted by the scientific establishment, this technology offers the prospect of a more economical and reliable method of maintaining photometric standards.

## 4. Spectral Sources and Pyrometry

While photometric units are integral measures over the whole spectrum, there is considerable value in standards and measurement services that provide spectral information too. Increasingly, detectors calibrated for spectral responsivity serve these needs [48]. However, in many applications standard sources remain indispensable. In this section, we provide two examples.

### 4.1 Thermal Sources for Spectral Radiometric Quantities and Radiation Temperature

In the last century, the most prevalent standard light sources were electric lamps. With reasonable care, they could be calibrated and shipped to customers and field sites, and they were easier to operate than the flame standards of earlier times.

NIST continues to supply a variety of lamps for different needs. Some lamps are calibrated for spectral irradiance, the light that the lamp casts on a surface a specific distance away. Other lamps are calibrated for spectral radiance. These have broad, flat filaments that one observes directly. But no matter what radiometric property a lamp is designed to communicate, the ultimate goal is to allow a customer to calibrate radiometric instrumentation for its intended function.

Until recently, the physical foundation for spectral radiance and irradiance standards was the predictable spectral output of blackbody sources that obeyed Planck’s radiation law [49, 50]. Of most importance were those blackbody sources that operated at the freezing-point temperatures of pure metals (that is, the temperature at which a molten metal solidified). One could compute the spectral radiance of these blackbodies, but only as well as you knew what their temperatures actual were.

In 1990 at NIST, Mielenz et al. determined the freezing-point temperature of gold to be 1337.33 K with an expanded relative uncertainty of 0.34 K (coverage factor *k* = 3) using a filter radiometer (FR) similar to that shown in Fig. 2 [51]. This achievement provided the impetus at NIST to decouple the spectral radiance and irradiance scales from any particular temperature scale (e.g., IPTS-68 or ITS-90) that might predefine the metal freezing-point temperatures. Instead, blackbody temperatures began to be measured *ab initio* using FRs traceable to absolute detectors [52–54]. Other national laboratories, including the National Physical Laboratory (NPL) in the U.K., the Physikalisch-Technische Bundesantalt (PTB) in Germany, and the All-Russian Institute for Optophysical Measurements (VNIIOFI) in Russia have also pursued this area of research. Early results were reported as part of an international intercomparison in 1998 [55].

**Fig. 14:**
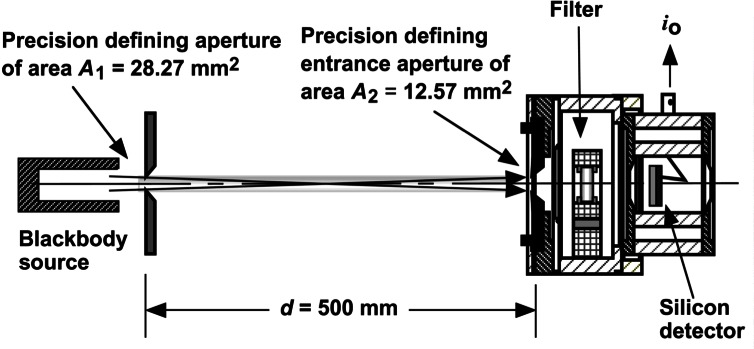
Schematic drawing (not to scale) of the experimental layout for the use of a filter radiometer in the measurement of the temperature of a blackbody source.

The basic technique for temperature measurements using an FR is shown in Fig. 14. A blackbody source at a temperature *T* is placed behind a precision circular aperture of known area *A*_1_ (radius *r*_1_) such that this aperture determines the amount of the source viewed by an FR positioned at a distance *d*. The FR has an aperture of known area *A*_2_ (radius *r*_2_) and has been calibrated such that its ASR, *s*(λ), is determined over the wavelength region where the silicon detector is responsive. The dimensions shown in the figure are typical in the NIST implementation. The spectral radiant flux Φ(λ) at the detector is given by Eq. (13) in terms of the spectral radiance *L*(λ) of the blackbody source and the geometrical parameters:

$$\begin{array}{l} \Phi \left(\lambda \right)=\frac{L\left(\lambda \right)A_{1}A_{2}}{D^{2}}\left(1+\delta +\cdots \right),\\ \text{where}\;D^{2}=\left(r_{1}^{2}+r_{2}^{2}+d^{2}\right),\;\text{and}\;\delta =\frac{r_{1}^{2}r_{2}^{2}}{D^{4}}. \end{array}$$ (13)

The expressions for *D* and δ arise from the finite sizes of the apertures, similar to the configuration factor in Eq. (12) [46, 47].

Analogous to the treatment above for the FR used as a photometer, we can write the output current *i*_o_ of the FR in terms of the ASR and the spectral radiant flux Φ(λ). Using the expression for spectral radiant flux from Eq. (13) we can write

$$\begin{array}{l} i_{o}=\int_{\lambda }\Phi \left(\lambda \right)s\left(\lambda \right)d\lambda ,\\ =\frac{A_{1}A_{2}}{D^{2}}\left(1+\delta +\cdots \right)\int_{\lambda }L\left(\lambda \right)s\left(\lambda \right)d\lambda . \end{array}$$ (14)

We can substitute into Eq. (14) Planck’s well-known expression for the spectral radiance from a blackbody source and get the explicit dependence between the output current of the FR and the temperature of the blackbody source. The result is given in the following, where *h* is the Planck constant, *c* is the speed of light in vacuum, *k* is the Boltzmann constant, and ε _λ_ is the emissivity of the blackbody cavity.

$$i_{o}=\frac{A_{1}A_{2}}{D^{2}}\left(1+\delta +\cdots \right)\int_{\lambda }\frac{2hc^{2}\varepsilon_{\lambda }}{\lambda^{5}\left(e^{\frac{hc}{\lambda kT}}-1\right)}s\left(\lambda \right)d\lambda$$ (15)

The importance of this equation is that it provides the relationship between the temperature being measured and the FR output, in a form that is dependent only upon known physical constants, geometric factors, and the ASR of the FR. Eq. (15) also indicates the important sources of uncertainty in the measurement. The largest component of the uncertainty usually arises from *s*(λ). This limiting factor, as in photometry, indicates the need for continued improvement in ASR determinations and improvements in the design of FRs. The defining apertures and distances are also important. However, the geometry of the experiment can usually be arranged such that the correction term δ is small and negligible. If the diameters of the apertures are small compared to the distance *d*, then *D* reduces to *d*, and the calculation is simplified considerably. The blackbody cavity must also be constructed such that the emissivity is a known quantity, which usually means the blackbody is designed to ensure the emissivity is nearly unity and independent of wavelength. The blackbody must be designed so the calculated emissivity can be checked experimentally.

**Fig. 15:**
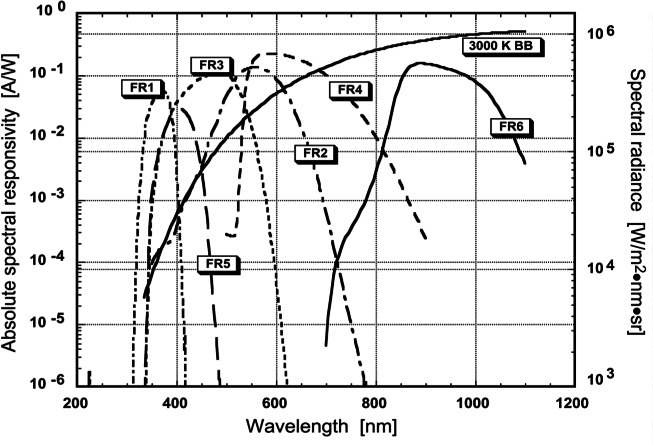
The absolute spectral responsivities of a number of filter radiometers used for temperature measurement at NIST. Also plotted for reference is the spectral radiance of a 3000 K blackbody source used as a spectral irradiance and radiance source. The horizontal scale is wavelength in nm, the left vertical scale is absolute spectral responsivity in A/W, and the right vertical scale is the spectral radiance of the blackbody source in W/(m^2^ nm sr).

Figure 15 shows the ASRs of a number of FRs constructed at NIST to be used in temperature determination. Also shown in the figure is a plot of the spectral radiance from a 3000 K blackbody, typical of those used as the basis for spectral irradiance or spectral radiance lamp calibrations. The spectral radiance increases by orders of magnitude at longer wavelengths, hence the responsivities of the FRs must be well characterized over all wavelengths where silicon has a response. Even small light-leakage of the filter in the longer wavelength region can introduce a significant contribution to the integral in Eq. (15).

The understanding of the out-of-band response of an FR is one of the driving forces for the construction of SIRCUS, discussed earlier in this paper. SIRCUS will allow the measurement of the ASR of an FR far from the central wavelengths of transmittance, spanning a dynamic range of up to 10 orders of magnitude. Through an accurate determination of the tail portions of the ASR, uncertainties in temperature measurement will be minimized.

**Fig. 16:**
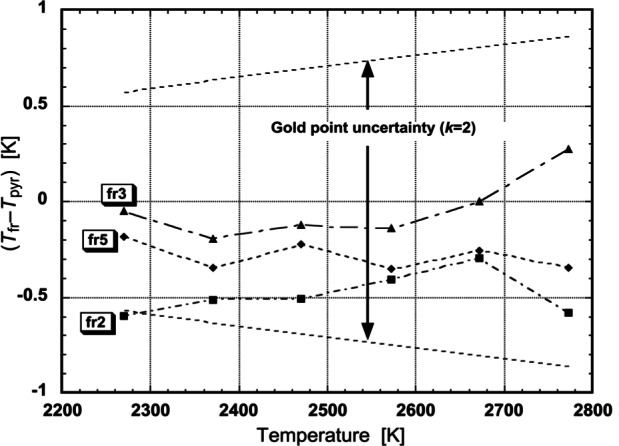
The agreement between pyrometry and the new filter radiometers. The horizontal scale is the temperature of a blackbody source in K, and the vertical axis is the difference in the determination of that temperature between the NIST pyrometer and three of the filter radiometers. Shown for reference are the uncertainties that would be expected using the pyrometer due to uncertainties in the NIST gold freezing-point determination.

The FRs whose ASRs are shown in Fig. 15 have been used to measure temperatures of a variable-temperature blackbody that was independently measured using the NIST photoelectric pyrometer [56]. The comparison of the results for three of the FRs is shown in Fig. 16. We see that the two methods gave the same temperatures to well within their combined experimental uncertainties. Of most significance was the uncertainty of the gold-point reference temperature, that is, gold’s freezing-point temperature as previously determined at NIST. The calibration chain for the pyrometer traced back to this key parameter, and the effect of its uncertainty at different temperatures is shown explicitly in the figure. However, the uncertainty of the gold-point temperature does not affect the FR method, thus demonstrating how FRs can improve radiation temperature metrology.

That said, the results using the other FRs were not comparable because of larger uncertainties in the determination of their ASRs or, as in the case of FR1, the diminished signal from the blackbody in the ultraviolet region of the spectrum. The uncertainties in the NIST measurement of ASRs are larger in the UV and near-IR (800 nm to 1200 nm) and hence the uncertainties when using FR1, FR4, and FR6 were larger than when using the other devices. However, as NIST improves the ASR measurements in the UV and near IR by the use of SIRCUS, and improves the transfer-standard detectors used in the DSC laboratories, the uncertainty in the radiation temperature measurement will decrease significantly.

We expect to routinely perform the characterization of FR ASR’s to within a relative standard uncertainty of 0.05 % using SIRCUS, which will provide the capability of determining temperature to within a relative uncertainty of about 0.1 % at 3000 K. This will improve the radiation temperature measurements discussed in Ref. [56], as well as the NIST scales of spectral irradiance and spectral radiance. Further, the pyrometers used for calibration of pyrometry lamps and other pyrometers can have their measurement scale set directly by viewing a blackbody that has been characterized with an FR system. When this is fully implemented in the pyrometry laboratory, we expect the uncertainty in the calibration of a pyrometer to decrease by a factor of two or more.

Once the temperature of a blackbody is determined, its spectral characteristics can be used to calibrate the spectral irradiance of lamps. This can be understood by using the definitions given above in Eq. (13). Using these we can rewrite an expression for the spectral irradiance *E*(λ)) for the configuration shown in Fig. 14:

$$E\left(\lambda \right)=\frac{\Phi \left(\lambda \right)}{A_{2}}=\frac{L\left(\lambda \right)A_{1}}{D^{2}}\left(1+\delta +\cdots \right).$$ (16)

NIST uses FEL quartz-halogen lamps as calibration artifacts in spectral irradiance scale dissemination to its customers. High-temperature blackbodies that can operate near the distribution temperature of the FEL lamps (2800 K to 3100 K) have been developed, and hence their irradiance can be compared directly to the test lamp for a direct calibration. Previous techniques starting out from the gold freezing point required several more steps in the calibration because the gold freezing point is at a much lower temperature than the distribution temperature of most lamps used for calibration devices [55, 57]. This disparity in signal levels in the visible wavelength region for calibration systems contributed significantly to the uncertainty in the calibrations for spectral irradiance and radiance.

**Fig. 17:**
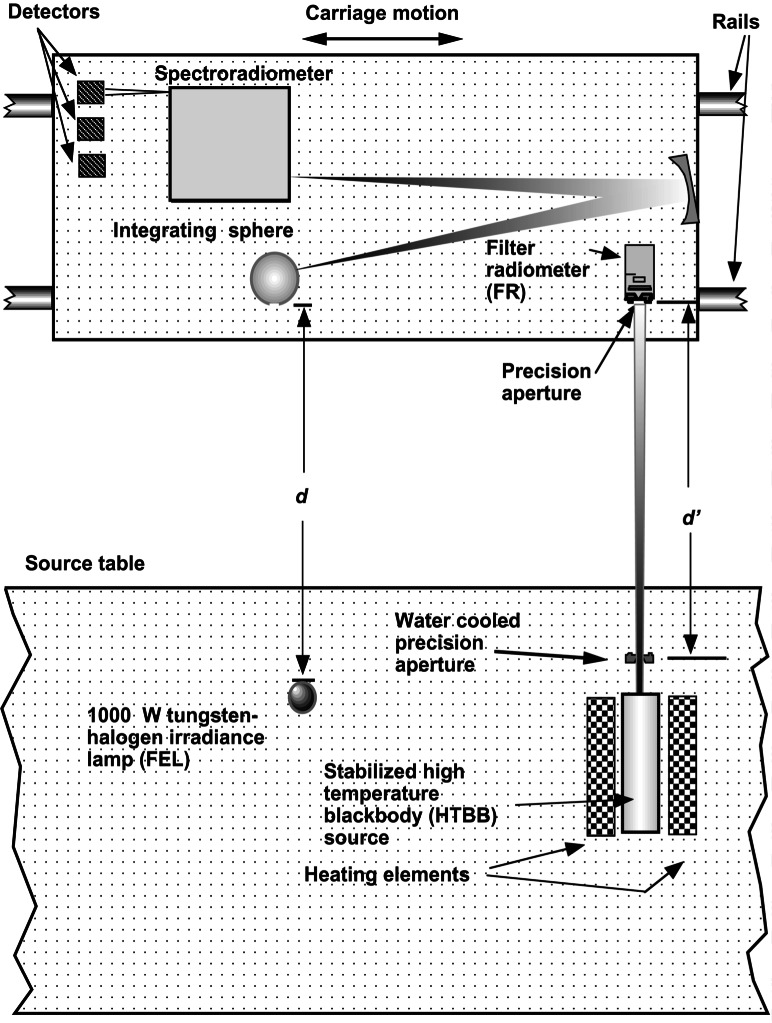
Schematic drawing (not to scale) of the instrument used for spectral irradiance measurement and realization of radiometric scales based upon known physical principles. The major components of the instrument are labeled in the figure.

Figure 17 shows the schematic layout for an instrument that determines the spectral irradiance of a test lamp using the method just described. The carriage is moved to place the FR in a position to view the output of a high-temperature blackbody (HTBB) source. Using Eq. (15) and measuring all the parameters allows the temperature of the HTBB, and thus its spectral radiance and irradiance to be deduced. The carriage then is moved so that the integrating sphere is in a position to view the HTBB. The system composed of the spectroradiometer, its input optics, and detector is calibrated from this known spectral radiance. The distance *d'* may differ in the placement of the integrating sphere and the FR because of their differing entrance apertures. An effort is made to have both of these devices view the same solid angle of the HTBB in order to eliminate problems from possible temperature variations within the HTBB cavity. The spectroradiometer has up to three different detectors that are chosen for optimal sensitivity in different portions of the wavelength region from 200 nm to 2500 nm. Once the spectral irradiance responsivity of the spectroradiometer system is determined, the carriage is moved to a position where the integrating sphere aperture is illuminated by a test lamp, in this case an FEL quartz-halogen lamp. The spectral irradiance of the test lamp is then measured with the calibrated instrument. In practice, to avoid the continual use of the HTBB, a set of working-standard lamps is calibrated utilizing the blackbody, and they in turn can be used to calibrate routine test lamps for customers. With a proper choice and selection of working standard lamps there is no significant increase in uncertainty as compared to direct use of the blackbody on each calibration.

The carriage can move the integrating sphere into positions to view up to six different sources, and hence several different lamps can be calibrated in a series. The FR can be positioned to view the lamps as well as the HTBB, which offers a way of monitoring the stability of the working standard lamps. This lamp monitoring is a component of the quality-control regimen to ensure a stable and reliable measurement system.

**Fig. 18:**
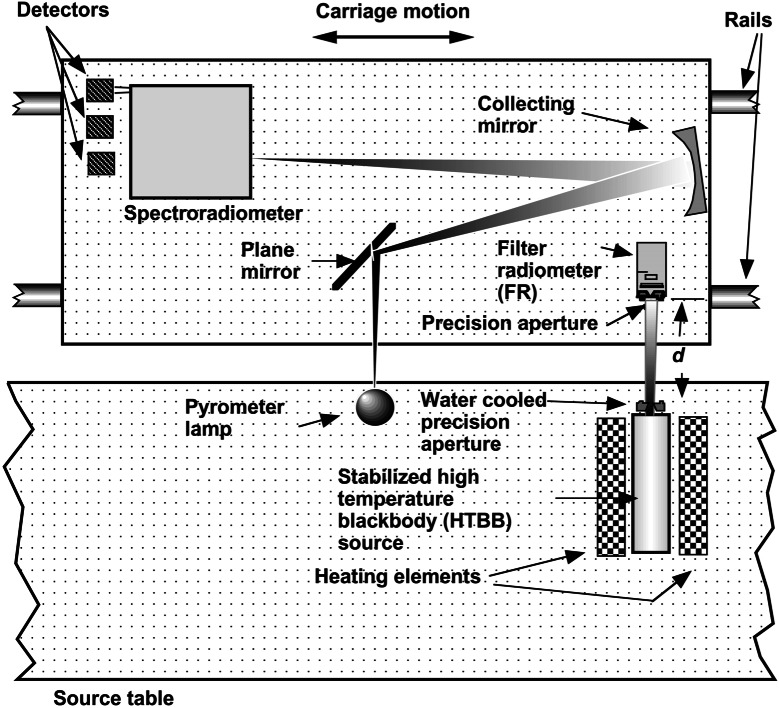
Schematic drawing (not to scale) of the instrument used for spectral radiance measurement and realization of radiometric scales based upon known physical principles. The major components of the instrument are labeled in the figure.

A slight adjustment of the input optics provides the capability of creating a spectral radiance scale based upon an HTBB. NIST can provide calibrations for spectral radiance in two general ways: on a specialized lamp calibrated for its output, and on a detector system calibrated for its spectral radiance responsivity. The configuration shown schematically in Fig. 18 indicates how the lamp calibration is performed. The FR is again used to determine the temperature, and thus the spectral radiance of the HTBB. The carriage then moves a plane mirror to a point where a portion of the HTBB source area is focussed onto the entrance slit of the spectroradiometer, and the spectral radiance responsivity of the spectroradiometer system is determined. Finally, the carriage is moved so that the calibrated spectroradiometer views a portion of the filament of a specialized radiance lamp.

Additionally, a second detector system can be mounted in a position equivalent to the FR shown in Fig. 18, and its spectral radiance responsivity determined by viewing either the HTBB or a calibrated lamp. Radiometers designed to measure spectral radiance generally have input optics that view a fixed solid angle. The calibration effort tries to have the test radiometer view the same source area that the reference spectroradiometer does in order to avoid errors due to any temperature gradients in the sources. These issues of having uniform sources and providing the same viewing area for the device under test and the reference spectroradiometer are central to decreasing the uncertainty of these calibrations.

The transition to the FR-based spectral source scales is taking place as this paper is being written in early 2000, and we expect it to be fully implemented along with a new Facility for Automated Spectroradiometric Calibrations (FASCAL) within a year. These changes will improve the uncertainties in the calibration of both lamps and pyrometers. Based upon our initial results, we expect the uncertainties to improve as shown in Table 2 for spectral irradiance and in Table 3 for spectral radiance. The uncertainties shown are a result of a detailed treatment of the measurement equation for the calibration, using the uncertainties of temperature determination that we have presently demonstrated with the FR technique. The uncertainties for the spectral quantities are the relative expanded uncertainties with a coverage factor of *k* = 2. These decreases in uncertainty represent a significant improvement for our customers. The use of higher-quality FRs offers a mechanism for additional improvement as the state-of-the-art in detector calibration improves. Other improvements, such as better variable-temperature blackbodies that could be used routinely, instead of lamps, could also decrease the uncertainties significantly.

### 4.2 Synchrotron Radiation

NIST has maintained a synchrotron radiation source since 1961, when a 180 MeV electron storage ring was commissioned as the Synchrotron Ultraviolet Radiation Source (SURF). It began the tradition at NIST of using synchrotron radiation to study a wide range of physical problems.

In 1974, SURF was upgraded to 240 MeV, with greater operating current. The original magnet was retained, but the rest of the major components were replaced or modernized. Major changes included the replacement of the old glass vacuum envelope with a stainless steel vacuum chamber and the introduction of a new injection system. This instrument was named SURF II, and it underwent a number of additional improvements that enhanced beam lifetime, beam current, and operating energy [58]. SURF II typically operated in a mode where the output radiant flux into an optical system could be known to within a relative standard uncertainty of about 2 %. This uncertainty, while appropriate for many uses, limited SURF II’s usefulness as an absolute radiometric source.

In 1994 a decision was made for a complete upgrade and redesign of SURF to create a new high-accuracy facility that is called SURF III. The major change effected in the upgrade was the replacement of the original 127 metric ton laminated iron magnet with a new 181 metric ton solid iron magnet. SURF I operated in a betatron mode and relied upon the laminated design to reduce electrical losses due to eddy currents. However, this design reduced the uniformity of the magnetic field, a major source of uncertainty.

**Fig. 19:**
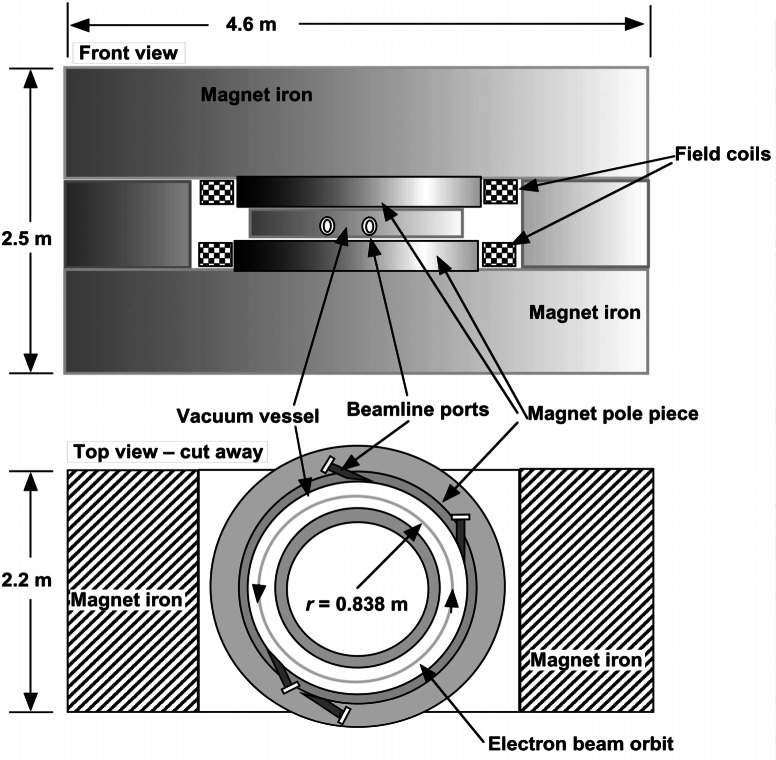
Schematic drawing of the magnet and vacuum chamber for Synchrotron Ultraviolet Radiation Facility (SURF) III.

A drawing of SURF III that shows its major features is shown in Fig. 19. Electrons are injected into the magnetic field at an energy of 10 MeV using a small microtron accelerator (not shown). The magnetic field bends the electrons into a circular orbit which passes through a radio-frequency (RF) cavity. The electric field in the cavity accelerates the electrons each time through. This, in combination with a ramp-up of the magnetic field (using the electromagnetic field-coils), maintains the electrons in a steady orbit. The process continues until the magnetic field reaches 1.4 T, which holds electrons with an energy of about 350 MeV [59]. At this point, an equilibrium is attained as the power supplied by the RF cavity balances the radiative synchrotron losses, discussed below. The electron beam orbits inside a toroidal ultrahigh-vacuum vessel that has viewports for a number of electron-beam tangent points, through which the synchrotron radiation exits the beam region for external use (not shown in detail). The field coils provide the magnetic field of the pole pieces, which have been machined to provide appropriate vertical and radial field patterns for focussing and containment of the electron beam. The rest of the iron serves as a magnetic return-loop for the field.

The electrons in SURF III move in a circular orbit, as shown in Fig. 19, due to the familiar Lorentz force on charged particles in a magnetic field. In 1898, Lienard showed that when a charged particle undergoes centripetal acceleration, such as in circular motion, it radiates electromagnetic radiation. The electrons in the SURF III storage ring are at highly relativistic energies, requiring relativistic electrodynamics to determine the spectral distribution of the radiation. The Schwinger theory shows that a narrow cone of light is emitted forward, in the electrons’ direction of motion, which contains very high harmonics of the orbital frequency [60]. At SURF III, the synchrotron radiation is emitted with significant intensity at wavelengths as short as 1 nm and at wavelengths up into the far-infrared and RF range. The vertical angular spread of the radiation is very narrow and forward at the shortest wavelengths, but diverges at longer wavelengths.

**Fig. 20:**
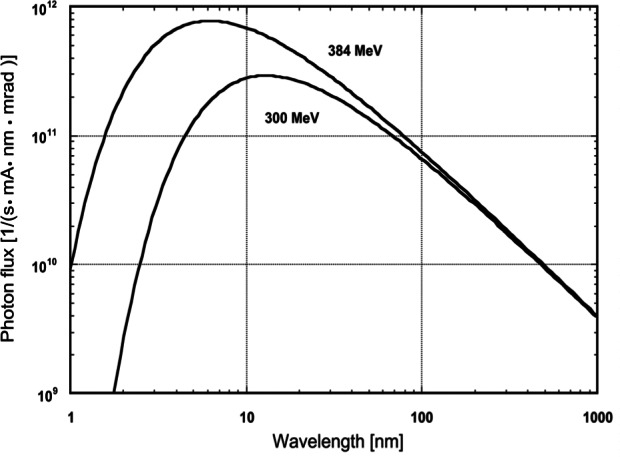
Plot of the flux of radiation from the Synchrotron Ultraviolet Radiation Facility (SURF) III calculated using Schwinger theory. The horizontal axis is logarithmic and is wavelength in units of nm. The vertical axis is logarithmic and shows the photon flux in units of numbers per second, wavelength interval, electron current, and horizontal viewing angle. The signal has been integrated over the vertical angle of emission.

Figure 20 shows the result of calculations using the Schwinger theory for two operating energies of SURF III. Increasing the energy of the stored beam increases the short wavelength output but does not substantially affect the long-wavelength output. The vertical scale in Fig. 20 is related to the radiant intensity of the beam per milliamperes of beam current. To calculate the radiance of SURF III it is necessary to also consider the spatial profile of the electron beam in orbit as well as other geometrical factors. For purposes here, the radiant intensity has been integrated over the vertical angle of emission, which at 100 nm is about 4 milliradians (full-width at half-maximum intensity). Depending upon the monochromator, the wavelength region utilized, and operating conditions, SURF III can deliver monochromatized photon fluxes of typically 10^10^ s^−1^ to 10^−13^ s^−1^ [61].

A cryogenic radiometer has been installed on a beamline at SURF III, and it will be used to calibrate detectors and filters and to develop procedures to calibrate the output of SURF III. Theory indicates that if the magnetic field, radius of the orbit, and electron current are known, then the absolute radiant intensity of the beam can be calculated. At SURF III we will exploit two different techniques to measure the electron current and hence determine the output of the storage ring. The first will be to use an FR as described previously, although instead of the Planckian spectral distribution we will have the distribution shown in Fig. 20 derived by Schwinger. By measuring the radiant flux in a specific solid angle with a well-characterized FR, the beam current can be directly determined if the magnetic field and radius of orbit are known. A second method depends onmeasuring the radiation from a very small number of electrons and deducing the radiant intensity per electron. Scaling this up to normal operating currents relies on the linearity of silicon detectors [62]. With the combinationof these methods SURF III’s radiant intensity can be predicted to within a relative uncertainty of 0.2 % to 0.5 %, depending upon wavelength and operating conditions.

With these innovations and improvements, SURF III serves as a primary optical power source from wavelengths as short as 1 nm to the far-infrared and terahertz region of the spectra. In addition to serving as a primary spectral source, the radiation from SURF III is employed in a variety of calibration programs for UV detectors, optical properties of materials, reflectometry in the soft x-ray region, and as a light source for a cryogenic radiometer program using synchrotron radiation [31].

## 5. Laser Power and Energy

In the early 1960s, as lasers were first being developed, NIST also began developing standards and measurement techniques required for this new technology. Lasers, as opposed to previous light sources, produced narrow beams with very high spatial and temporal co-herence, an extremely nonuniform irradiance profile (which was nonetheless significant in its detail), and in some cases, unprecedented irradiance or energy density levels, especially in short pulses. These were the properties that made lasers useful for many novel applications, but the traditional methods of radiometry were unable to measure them accurately and consistently.

**Fig. 21:**
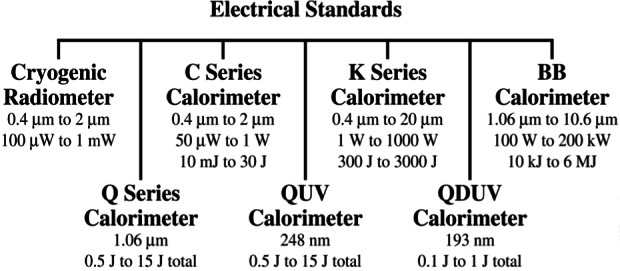
NIST primary standards for laser power and energy. Different calorimeters or radiometers are used in different wavelength and power or energy regimes.

Over the years, NIST has developed a substantial portfolio of standards and measurement services to characterize laser radiation at all commonly used wavelengths and over a wide range of powers and energies. The principal standards now in use (Fig. 21) are electrically calibrated calorimeters and radiometers. They accurately compare the power or energy in laser radiation to electrical power or energy, thus providing traceability to SI units.

The first NIST laser-energy standard was a “liquid-cell calorimeter,” developed by Jennings in the early- to mid-1960s [63]. This calorimeter used an aqueous solution of CuSO_4_ in a cell with a quartz window. A small amount of dark ink was added to the solution to increase its wavelength-absorption range. Thermocouples measured a temperature rise caused by laser-light absorption as well as the corresponding temperature rise caused by an electrical heater. Through equivalence, this device measured the energy of individual laser pulses. Although designed for measuring the energy from pulsed lasers, such as ruby and Nd:glass varieties, the liquid cell calorimeters also worked well for measuring the average power from cw laser sources. For a number of years, these liquid cell calorimeters were used as primary standards at NIST and furnished to DoD primary standards laboratories for calibrating their laser power and energy meters.

**Fig. 22:**
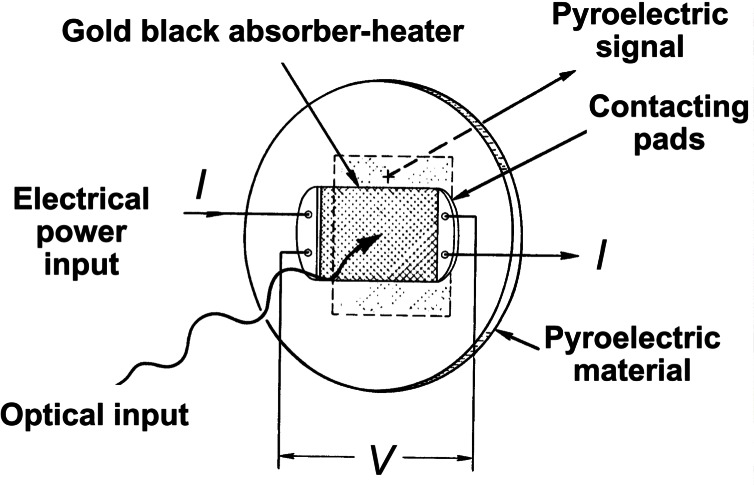
Electrically calibrated pyroelectric radiometer. Optical input generates a pyroelectric signal from a pyroelectric material behind the receiver surface shown. Electrical heating can also be applied through current leads shown. Applied electrical power is current *I* times voltage drop *V*.

Also during the early years of laser-power measurements, Phelan, Cook, Hamilton, and Day developed the electrically calibrated pyroelectric radiometer (ECPR) as a laser power standard [64–66]. The basic elements are shown in Fig. 22. A thin sample of pyroelectric material is sandwiched between two conducting electrodes. A pyroelectric material contains a permanent electric-dipole moment, or polarization field, that varies as a function of temperature. Thus, when it is heated, the electrical potential across the crystal changes. This type of instrument is sensitive to changes in temperature (rather than to the absolute temperature) because the changing surface charges on the crystal are rapidly equalized by free charges attracted to the electrodes.

Here again, heating power can be applied either optically or electrically. The front electrode is coated with a gold-black that is both highly absorbing of laser radiation and somewhat electrically conductive. During operation, electrical heating is alternated with optical heating at about 15 Hz, and the electrical power is adjusted to obtain equivalence with the laser radiation. Because of its sensitivity and other advantageous properties, this device was commercialized, and it is in widespread use in laboratories throughout the world.

In the early 1970s, West developed a cavity-based calorimeter whose responsivity could be analyzed with well-established isoperibol (constant temperature environment) techniques [67, 68]. Various models of isoperibol calorimeters have since been developed, and today they are used as primary standards for many NIST laser-measurement services (Fig. 21). For the best accuracy in low-power laser measurements, NIST also uses a laser-optimized cryogenic radiometer (LOCR) [69] whose operation is similar to the HACR described earlier. However, the isoperibol calorimeters are used for higher powers, for pulsed lasers, and in other situations where comparison to the LOCR scale is not advantageous.

Both the LOCR and the isoperibol calorimeters contain cylindrical cavities that absorb laser radiation. Thermal sensors attached to the absorbing structure monitor the resulting temperature increase. A temperature-stabilized enclosure or jacket provides the environment required for isoperibol conditions, which allows the temperature sensors to accurately indicate the quantity of laser radiation received. As with the ECRs discussed earlier in this paper, electrical substitution provides the connection between optical power and the NIST electrical measurement scales.

The basic principles of cryogenic radiometers for power measurement were reviewed earlier in this article. Here, we explain the behavior and operation of the isoperibol calorimeters for energy measurement.

**Fig. 23:**
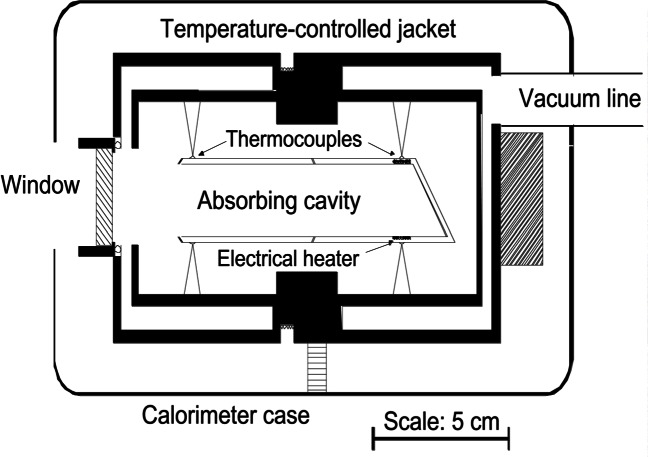
Isoperibol calorimeter. Laser radiation absorbed by the central cavity causes temperature of themocouples to rise. Electrical heating can also be applied to calibrate the thermocouple response. The system is well isolated from its environment.

**Fig. 24:**
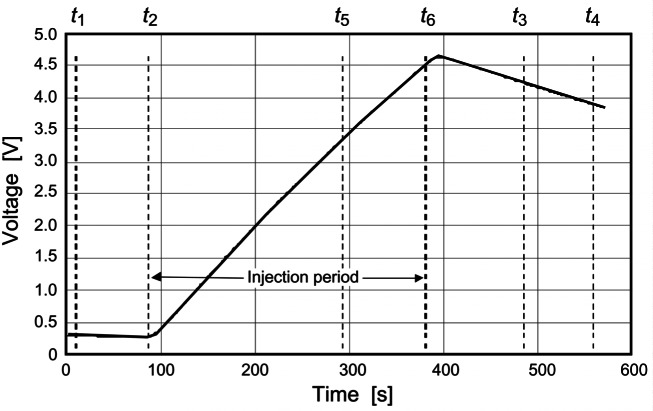
Voltage vs. time output from isoperibol calorimeter. Horizontal in seconds shows events in the measurement cycle, such as the period of laser irradiation.

A cross section of a typical isoperibol calorimeter is shown in Fig. 23 [70]. Its relatively simple geometry allows a straightforward analysis of its response to a laser-energy pulse [71, 72]. The energy *E* absorbed by the calorimeter is related to voltage time-response *V*(*t*) of the temperature sensors,

$$E=K_{e1}\left[V_{f}-V_{i}-\varepsilon \int_{t_{i}}^{t_{f}}\left(V\left(t\right)-V_{\infty }\right)dt\right],$$ (17)

where *K*_el_ is a constant determined by electrical-energy substitution; *V*_i_ and *V*_f_ are voltages that occur at times *t*_i_ and *t*_f_, respectively, that temporally bracket the laser-injection period; *V*∞ is the steady state or convergence voltage; ε and is the cooling constant described below. This equation essentially restates the first law of thermodynamics: the energy *E* absorbed is equal to the change in internal energy [*K*_el_ times (*V*_f_ − *V*_i_)], minus the heat transferred from the system during the measurement (*K*_el_ε times the voltage integral between times *t*_i_ and *t*_f_). The quantity in brackets is referred to as the corrected voltage rise Δ*V* and represents the output of the calorimeter for an individual energy measurement. Figure 24 shows a typical voltage-response curve.

This equation assumes a linear system in which both heat capacity and thermal conductivity are constant. To achieve this condition as closely as possible, the energy injection time is limited to keep the temperature rise of the cavity to less than a few degrees. As a linear system, the output response can be represented by a series of exponential terms, but before and sometime after the energy deposition, the voltage response approximates a single exponential function containing the cooling time-constant ε.

The electrical calibration factor *K*_el_ is determined by depositing a known amount of electrical energy *E*_el_ through a heater thermally bonded to the absorbing cavity. Using the relationship

$$K_{\text{el}}=\frac{E_{\text{el}}}{\Delta V},$$ (18)

*K*_el_ is determined for each calorimeter. Obviously, this implies that a calorimeter behaves the same for electrical-energy deposition as it does for laser-energy absorption. In reality, the temperature gradients within the calorimeter are different in the two cases, and consequently their voltage responses may differ as well. This electrical-optical inequivalence is minimized by careful design of the calorimeter. For example, in the calorimeter in Fig. 23, the heat (no matter where it originates in the innermost cavity) is forced to travel through a common isothermal element connecting the two inner cylindrical cavities before arriving at the temperature sensors on the outer cavity. This inequivalence can also be reduced by using cryogenic temperatures, as is done with cryogenic radiometers. However, for room temperature devices it remains a major source of uncertainty in the measurement.

Since not all the laser energy incident on the calorimeter causes heating, the actual measurement equation is

$$E=\frac{K_{\text{el}}\Delta V}{\tau \alpha },$$ (19)

where τ is the transmittance of the calorimeter window (unity when no window is used) and *α* is the absorptance of the cavity. This absorptance factor accounts for light scattered back out of the calorimeter as well as radiative emission due to local heating. Indeed, thermal radiation is generated primarily where the cavity is the hottest, in the neighborhood where the laser beam is absorbed. This heat pattern is generally not replicated through electrical substitution, potentially causing an inequivalence condition. However, to minimize such problems and to prevent damage to the cavity surfaces, for high-power laser measurements the laser beam is first reflected from a curved surface to spread its energy over a larger area. By careful application of optical spreading techniques, isoperibol calorimeters have been developed for power measurements at the kilowatt [73] and multi-kilowatt [74, 75] levels.

If the incident laser-irradiance level is below 200 W/cm^2^ then most black coatings (e.g., black paint, copper oxide, etc.) work fine and are not likely to suffer damage. However, pulsed laser radiation presents a problem. The energy from short pulses, such as those produced by *Q*-switched or mode-locked lasers, is deposited in a time interval too short for the heat to diffuse away from the absorption area. The resulting localized temperature increase can exceed the damage threshold of the absorbing material. Consequently, for calorimeters designed for measuring lasers with pulse-energy densities exceeding 300 mJ/cm^2^, an absorbing volume is typically used instead of an absorbing surface. Typical volume-absorbing materials include liquids (as in the liquid-cell calorimeter discussed above), glasses, and certain ceramics.

NIST has designed and built several models of isoperibol calorimeters that use absorbing glasses for pulsed laser radiation. The absorption profile in these glasses can be expressed as

$$E\left(x\right)=E_{0}e^{-\alpha x},$$ (20)

where *E*(*x*) is the energy per area at a depth *x* into the material, *E*_0_ is the energy per area at the incident surface, and α is the absorption coefficient of the material at the particular laser wavelength being used. The maximum associated temperature rise (assuming no heat transfer during the pulse duration) is then given by

$$\Delta T=\frac{\alpha E_{0}}{\rho c},$$ (21)

where Δ*T* is the induced temperature rise at the material’s surface, *ρ* is the absorber density, and *c* is the absorber specific heat. Materials with a low value of α have a lower associated Δ*T* but require a greater thickness to absorb all the laser energy. Materials with a high value of have a α higher Δ*T* but require thinner samples. As a compromise between damage-threshold levels and desirable heat diffusion characteristics, the absorber material is chosen such that its inherent value of α allows complete absorption within a thickness of 1 mm to 2 mm. Using this guideline, glass absorbers have been applied to infrared [76] and ultraviolet [77, 78] laser measurements. Even though volume-absorbing materials minimize thermal damage, materials used in calorimeters must also be free from other effects, such as fluorescence and degradation, when exposed to laser pulses.

**Fig. 25:**
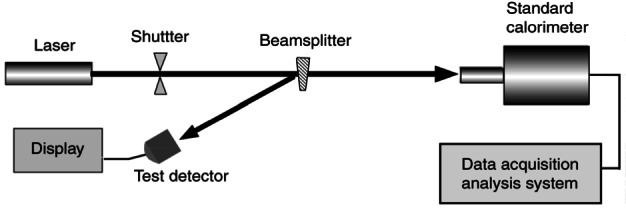
Beamsplitter-based laser power and energy measurement system. A beamsplitter allows a test detector to be calibrated in terms of a standard calorimeter.

When comparing other detectors to the laser power and energy standards (e.g., during detector calibration), various types of beamsplitter systems are frequently used. They allow simultaneous measurement of the laser power or energy by two or more detectors and, as a result, help to minimize the effects of laser-amplitude variations. Figure 25 illustrates the basic concept. Note that beamsplitters usually have a wedge geometry to eliminate interference effects.

Beamsplitters also extend the operating power range of the standards. In power measurement systems, each beamsplitter is used either to sample part of the beam for a monitor detector or it is used as a calibrated standard itself. When used as a standard, the ratio of powers in the two beams is determined as follows. First, two detectors (usually standard calorimeters) are placed in the beams. Their power ratio is measured a number of times and averaged as *R*_1_. The two detectors are then interchanged, and the ratio of power in the two beams is measured again to get an average *R*_2_. The absolute ratio is taken to be

$$R=\sqrt{R_{1}R_{2}.}$$ (22)

In this procedure, systematic effects cancel if the detectors are linear and spatially uniform. Once *R* is determined, other detectors can be placed in the beams and compared directly.

**Fig. 26:**
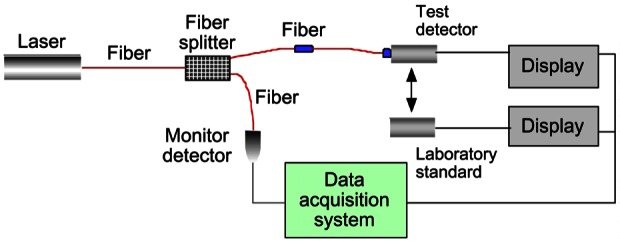
Optical fiber power measurement system. A fiber splitter allows a test detector to be calibrated in terms of a laboratory standard. A monitor detector ensures system stability.

For optical fiber power measurements, the beamsplitter system is modified to incorporate fiber components such as optical fibers and fiber splitters (Fig. 26). Laser radiation, typically at low power in the infrared wavelength region, is delivered to the detectors through optical fibers and specialized connectors. Due to the many reflecting surfaces inherent in fiber systems, the coherence of laser radiation can lead to interference effects and power instability if the measurement system is not designed properly [79].

Today, NIST is working to extend its measurement capability and improve the measurement uncertainty in existing laser-measurement areas. In addition, we are developing new capabilities to meet the emerging needs of the laser community. For example, to support the next generation of high-resolution semiconductor photolithography systems, calorimeter design efforts are in progress to develop standards for excimer-laser measurements at 157 nm. In addition to the power and energy measurements discussed above, related laser measurement capabilities are also maintained. These include source-based measurements such as irradiance profile [80, 81] and relative intensity noise (RIN) [82, 83], as well as detector-based measurements such as spatial uniformity [84], power linearity [85], spectral responsivity [86], frequency response [87], and impulse response. Also, various laser-specific materials issues are studied [88]. This combined capability allows NIST to give comprehensive coverage to the metrology needs of the laser community. As lasers with new wavelengths or power characteristics are employed by industry, NIST will continue to respond with useful standards and measurement techniques.

## 6. Conclusions

The redefinition of the candela in terms of optical power by the CPGM, and the adoption by the CIPM of the CIE *V*(λ) function for human visual response, has had a profound impact upon photometry and radiometry. As was intended, this definition shifted the emphasis in photometry and radiometry away from reliance upon blackbody sources operating at metal freezing-points to the reliance upon well-characterized optical radiation detectors.

This review has focussed on the impact of this new direction upon NIST activities, but the same basic story applies to major national metrology institutes around the world. While the redefinition pertained only to photometry, the impetus to develop better optical detectors has benefited the scientific disciplines of spectral radiometry and pyrometry as well. Over the same timespan that photometry has became dependent upon improved optical detectors, the use of lasers in communications and information technologies has also spurred the improvement in electrical-substitution radiometers used for laser-power measurements.

The real beneficiaries, however, are the customers of NIST calibration services, as the calibration ranges and uncertainties have improved significantly. Further, the stability of the measurement chain based upon the cryogenic radiometer provides a new level of robustness and continuity to the national measurement system. Customers, be they engaged in photography, display technology, electronics, optoelectronics, scientific instruments, aerospace, remote sensing, or other fields, all enjoy these benefits. They translate into new capabilities for process control, simplified measurement tools, and improved product quality. Indeed, the ongoing shift towards detector-based methods provides widespread gains in accuracy, efficiency, and stability.

## 7. Symbols

*c*: Heat capacity (specific heat) (J/K)
*d*: Distance (e.g., separation) (m)
*i*: Current (electrical) (A)
*i*_o_: Current, output (A)
*r*: Distance (e.g., radius or separation) (m)
*t*: Time (s)
*x*: Distance (e.g., depth) (m)
*s*_v,f_: Luminous flux responsivity (A/lm)
*s*_v,i_: Illuminance responsivity (A/lx)
*A*: Area (e.g., of an aperture) (m^2^)
*E*: Energy (J)
*E*: Energy density (J/m^2^)
*E*_v_: Illuminance (lx)
*G*: Thermal conductance (W/K)
*I*_v_: Luminous intensity (cd)
*K*_el_: Electrical calibration factor (J/V)
*K*_m_: Maximum spectral efficacy of radiation for photopic vision (683 lm/W)
*L*_v_: Luminance (cd/m^2^)
*R*: Resistance (electrical) (Ω)
*R*: Ratio (e.g., power)
*T*: Temperature (K)
*T*_0_: Temperature, reference (K)
*V*: Voltage (V)
*s*(λ): Absolute spectral responsivity (A/W)
*s*(555): Absolute spectral responsivity at 555 nm(A/W)
*s*_n_(λ): Relative spectral responsivity (normalized to 1 at 555 nm)
*E*(λ): Spectral irradiance [W/(m^2^•nm)]
*L*(λ): Spectral radiance [W/(m^2^•sr nm)]
*V*(λ): Spectral luminous efficiency function for photopic vision (originally, the Visibility function)
α: Absorptance (e.g., cavity); also:
α: Adsorption coefficient (m^−1^)
ε: Cooling constant (s^−1^)
λ: Wavelength (nm)
*ρ*: Reflectance (e.g., cavity or window); also:
*ρ*: Density, absorber (m^−3^)
τ: Transmittance (e.g., window)
Φ: Flux, optical (or radiant) (W) (historically, Φ_e_)
Φ_v_: Luminous flux (lm)
Φ_0_: Flux, incident optical (W)
(λ): Spectral flux, optical (or radiant) (W/nm)

## 8. Acronyms

ASR: Absolute spectral responsivity
CGPM: Conférence Générale des Poids et Mesures (General Conference on Weights and Measures)
CIE: Commission Internationale de l’Éclairage (International Commission on Illumination)
CIPM: Comité International des Poids et Mesures (International Committee on Weights and Measures)
DoD: Department of Defense (U.S.)
DSC: Detector spectral comparator
ECPR: Electrically calibrated pyroelectric radiometer
ECR: Electrically calibrated radiometer
ESR: Electrical substitution radiometer
FASCAL: Facility for Automated Spectroradiometric Calibrations
FR: Filter radiometer
HACR: High-accuracy cryogenic radiometer (NIST)
HTBB: High-temperature blackbody
IR: Infrared
LOCR: Laser-optimized cryogenic radiometer (NIST)
NBS: National Bureau of Standards
NPL: National Physical Laboratory (UK)
PTB: Physikalisch-Technische Bundesantalt
RF: Radio-frequency
RIN: Relative intensity noise
SI: Système international d’unités (International system of [metric] units)
SIRCUS: Spectral Irradiance and Radiance Calibrations using Uniform Sources
SURF: Synchrotron Ultraviolet Research Facility
TSD: Transfer standard detector
UV: Ultraviolet
VNIIOFI: All-Russian Institute for Optophysical Measurements

## Figures and Tables

**Table 1:** Corresponding radiometric and photometric quantitites. The radiometric quantities, on the left, are shown with their usual symbols and SI units of measure. When integrated spectrally using *V*(λ) weighting, one obtains the photometric counterpart, on the right

Radiometric quantity	Symbol	Unit	Unit	Symbol	Photometric quantity
Radiant energy	*Q*	J	lm s	*Q*_v_	Luminous energy
Radiant flux (power)	*P*, Φ	W	lm	Φ_v_	Luminous flux
Irradiance	*E*	W/m^2^	lm/m^2^=lx	*E*_v_	Illuminance
Radiance	*L*	W/(m^2^ sr)	lm/(m^2^ sr)	*L*_v_	Luminance
Radiant intensity	*I*	W/sr	lm/sr=cd	*I*_v_	Luminous intensity

J=joule, lm=lumen, s=second, W=watt, m=meter, sr=steradian, cd=candela.

**Table 2:** The present and expected relative expanded uncertainties (coverage factor *k* = 2) for calibrations of spectral irradiance *E*(λ) at typical wavelengths

Wavelength (nm)	Present expanded uncertainty (%)	Expected expanded uncertainty (%)
250	1.8	1.0
350	1.1	0.6
655	0.9	0.5
900	1.1	0.5
1600	1.4	0.5
2400	4.4	1.1

**Table 3:** The present and expected relative expanded uncertainties (coverage factor *k* = 2) for calibrations of spectral radiance *L*(λ) at typical wavelengths

Wavelength (nm)	Present expanded uncertainty (%)	Expected expanded uncertainty (%)
225	1.5	1.2
250	1.3	0.8
350	1.0	0.6
650	0.6	0.3
900	0.6	0.3
1550	0.5	0.3
2400	0.4	0.3

## Footnotes

1 In traditional notation, a subscript of λ on radiometric quantities indicates their functional dependence on wavelength (e.g., *E* for spectral irradiance and *V* λ for the visibility function). In this paper, we use modern notation (e.g., *E*(λ) and *V*(λ), respectively).

2 The fact that 540 × 10^12^ Hz does not correspond exactly to a wavelength of 555 nm in air causes for factor *K*_m_ to be slightly larger than 683 lm/W. See Ref. [2] for details. For the purpose of this paper, the differences are insignificant.
